# EP300-ZNF384 transactivates IL3RA to promote the progression of B-cell acute lymphoblastic leukemia

**DOI:** 10.1186/s12964-024-01596-9

**Published:** 2024-04-02

**Authors:** Zhijie Hou, Yifei Ren, Xuehong Zhang, Dan Huang, Fanzhi Yan, Wentao Sun, Wenjuan Zhang, Qingqing Zhang, Xihui Fu, Zhenghui Lang, Chenyang Chu, Boyang Zou, Beibei Gao, Bilian Jin, Zhijie Kang, Quentin Liu, Jinsong Yan

**Affiliations:** 1 Department of Hematology, Liaoning Medical Center for Hematopoietic Stem Cell Transplantation, the Second Hospital of Dalian Medical University, Dalian, 116027 China; 2 Liaoning Key Laboratory of Hematopoietic Stem Cell Transplantation and Translational Medicine, Dalian Key Laboratory of hematology, Diamond Bay institute of hematology, Blood Stem Cell Transplantation Institute, the Second Hospital of Dalian Medical University, Dalian, 116027 China; 3 Department of Pediatric, Pediatric Oncology and Hematology Center, the Second Hospital of Dalian Medical University, Dalian, 116027 China; 4 Institute of Cancer Stem Cell, Cancer Center, Dalian Medical University, Dalian, 116044 China; 5 Center of Genome and Personalized Medicine, Institute of Cancer Stem Cell, Dalian Medical University, Dalian, 116044 China; 6 Department of Pathology, Dalian Medical University, Dalian, 116044 China

**Keywords:** EP300-ZNF384, IL3RA, IL-3, B-cell acute lymphoblastic leukemia, Leukemogenesis

## Abstract

The *EP300-ZNF384* fusion gene is an oncogenic driver in B-cell acute lymphoblastic leukemia (B-ALL). In the present study, we demonstrated that EP300-ZNF384 substantially induces the transcription of *IL3RA* and the expression of IL3Rα (CD123) on B-ALL cell membranes. Interleukin 3 (IL-3) supplementation promotes the proliferation of *EP300-ZNF348*-positive B-ALL cells by activating STAT5. Conditional knockdown of *IL3RA* in *EP300-ZF384*-positive cells inhibited the proliferation in vitro, and induced a significant increase in overall survival of mice, which is attributed to impaired propagation ability of leukemia cells. Mechanistically, the EP300-ZNF384 fusion protein transactivates the promoter activity of *IL3RA* by binding to an A-rich sequence localized at -222/-234 of *IL3RA*. Furthermore, forced *EP300-ZNF384* expression induces the expression of IL3Rα on cell membranes and the secretion of IL-3 in CD19-positive B precursor cells derived from healthy individuals. Doxorubicin displayed a selective killing of *EP300-ZNF384-*positive B-ALL cells in vitro and in vivo. Collectively, we identify *IL3RA* as a direct downstream target of EP300-ZNF384, suggesting CD123 is a potent biomarker for *EP300-ZNF384*-driven B-ALL. Targeting CD123 may be a novel therapeutic approach to *EP300-ZNF384*-positive patients, alternative or, more likely, complementary to standard chemotherapy regimen in clinical setting.

**Supplementary Information:**

The online version contains supplementary material available at 10.1186/s12964-024-01596-9.

## Background

The *EP300-ZNF384* fusion gene is a common genomic lesion in patients with acute B lymphoblastic leukemia (B-ALL) and B/M mixed phenotype acute leukemia (MPAL) [1, 2]. It is generated by the t(12; 22) (p13; q13) chromosome translocation fusing *EP300* to *ZNF384* [1]. In B-ALL, *ZNF384* can fuse with 22 partner genes, including *EP300*, *TCF3*, *TAF15*, and *CREBBP* [3]. Among these, *EP300* is recognized as the most common fusion partner of *ZNF384*, found in approximately 3.7% of patients with B-ALL [4]. Most *EP300-ZNF384*-positive cases showed a typical immunophenotype (weak or absent expression of CD10 and aberrant expression of at least one myeloid antigen; CD13 or CD33) [5, 6]. Patients with *EP300-ZNF384* achieve more favorable clinical outcomes than those patients without it [5, 7].

In comparison to B-ALL with other genomic lesions, *EP300-ZNF384*-positive B-ALL is characterized by a distinct gene expression signature predominantly enriched in the upregulation of Janus kinase/signal transducers and activators of transcription (JAK/STAT) and cell adhesion pathways, and downregulation of the cell cycle and DNA repair pathways [7, 8]. Expression of *EP300-ZNF384* blocked the differentiation of mouse Lin^−^ c-Kit^Low^ cells into CD19^+^ B cells in vitro [9]. Mice transplanted with *EP300-ZNF384*-expressing bone marrow (BM) cells develop acute monoblastic leukemia with defects in B cell development, characteristic leukocytosis, anemia, splenomegaly, and accumulation of blast cells in the BM [9]. Expression of *EP300-ZNF384* in pro-B cells resulted in the development of pre-B cell ALL and differentiation arrest at the pre-B stage in a mouse transplantation model [10]. These results indicated that *EP300-ZNF384* is an oncogenic driver of B-ALL. Emerging studies have demonstrated that *EP300-ZNF384* promotes B cell proliferation, inhibits the differentiation of B cell, and maintains the hematopoietic stem cell (HSC) signature by activating the expression of *CLCF, BTLA, GATA3, ID2*, and *SALL4* [6, 11, 12]. However, the oncogenic mechanisms underlying the functions of *EP300-ZNF384* remain largely unknown.

IL3Rα (also known as CD123) is the α subunit of the interleukin 3 (IL-3) heterodimeric cell-surface receptors, which is widely expressed in hematopoietic stem/progenitor cells, monocytes, megakaryocytes, B-lymphocytes, and plasmacytoid dendritic cells [13–15]. Upon binding IL-3, IL3Rα recruits the βc subunit to form a functional IL3R complex, which regulates the proliferation, survival, and differentiation of hematopoietic cells [16–19]. IL3Rα is highly expressed in various types of leukemia, such as acute myeloid leukemia (AML), B-ALL, and blastic plasmocytoid dendritic neoplasms and has been recognized as a marker for leukemic stem cells in AML [20–23]. Elevated expression of IL-3Rα is associated with enhanced blast proliferation and a poor prognostic phenotype in patients with AML, indicating that CD123 is a promising therapeutic target for leukemia therapy [24–26]. Multiparameter immunophenotypic analysis demonstrated that patients with *ZNF384* rearrangements, including *EP300-ZNF384*, had significantly higher CD123 than those without a *ZNF384* fusion, suggesting that CD123 is a biomarker for *ZNF384*-related fusion [27]. These observations suggest the possible involvement of the IL3RA signal in *EP300-ZNF384*-primed B-ALL.

In this study, we investigated the functional roles of IL3RA signaling pathway in *EP300-ZNF384*-induced leukemogenesis and identified the regulatory mechanism of EP300-ZNF384 fusion protein that is responsible for *IL3RA* transcriptional activation. We further evaluated therapeutic potential of doxorubicin in *EP300-ZNF384* positive B-ALL cells in vitro and in vivo, pointing to that targeted therapies involving anthracyclines may produce a favorable outcome in patients with *EP300-ZNF384*.

## Materials and methods

### Sample preparation and cell cultures

Primary samples were collected from patients diagnosed with B-ALL, according to the French-American-British classification, at the Department of Hematology of the Second Hospital of Dalian Medical University. Peripheral blood mononuclear cells (PBMCs) were obtained from healthy individuals for BM transplantation and were used to purify CD19^+^ cells by EasySep™ Human CD19 Positive Selection Kit II (Cat. 17,854, Stemcell Technologies, Vancouver, BC, Canada) according to the manufacturer’s instructions. All patients and donors provided written consent for the protocols, which were approved by the Institutional Review Board and Medical Science Ethics Committee of Dalian Medical University in accordance with the Declaration of Helsinki (2023-XWLW NO.2). HEK-293T cells were purchased from the American Type Culture Collection. Human B-cell acute lymphoblastic leukemia cell lines NALM-6 and BALL-1 were purchased from iCell Bioscience Inc. (Shanghai, China). The human leukemia cell line KG-1α and the Burkitt’s lymphoma cell line Daudi were obtained from Professor Ying Lu (Institute of Dermatology, Xinhua Hospital, School of Medicine, Shanghai Jiao Tong University, China). Primary PBMCs were cultured in Iscove’s modified Dulbecco’s media (IMDM) supplemented with 20% fetal bovine serum (FBS, Gibco, Waltham, MA, USA). HEK-293T cells were cultured in DMEM supplemented with 10% FBS (Gibco). NALM-6, BALL-1, and Daudi cells were cultured in RPMI 1640 media supplemented with 10% FBS (Gibco). KG-1α was cultured in IMDM media supplemented with 20% FBS (Gibco). All cells were maintained in a humidified 5% CO_2_ atmosphere at 37 °C.

### Reagents and antibodies

Antibodies against ZNF384 (Cat. Ab176689; Abcam, Cambridge, UK), p-STAT5 (Cat. 9314; CST, Danvers, MA, USA), STAT5 (Cat. 94,205; CST), FLAG (Cat. F1804; Merck, Darmstadt, Germany), and GAPDH (Cat. 60004-1-Ig; Proteintech Group, Rosemont, IL, USA) were used for western blot analysis. The following antibodies were used for flow cytometry analysis: APC mouse anti-human CD123 (Cat. 560087; BD Biosciences, Franklin Lakes, NJ, USA), APC mouse IgG2a, κ Isotype Control (Cat. 555576; BD Biosciences), APC Mouse Anti-Human CD19 (Cat. 340437; BD Biosciences), and an IgG1 kappa isotype control (Cat. 340442, BD Biosciences). The doxycycline hyclate was purchased from Meilunbio (Cat. MB1088; Dalian, China). Recombinant human IL-3 was purchased from PeproTech (Cat. 200-03; Cranbury, NJ, USA). Doxorubicin hydrochloride (Cat. T1020), dexamethasone (Cat. T1076) and vincristine sulfate (Cat. T1270) were purchased from TOPSCIENCE (Singapore). Puromycin was purchased from Selleck Chemicals (Cat. S7417; Houston, TX, USA). Enzyme-linked immunosorbent assay (ELISA) kit for IL-3 was purchased from Cloud-Clone Corp. (Cat. SEA076Hu; Houston, TX, USA). Pegaspargase was purchased from Jiangsu Hengrui Pharmaceutical Co., Ltd.

### RNA library preparation, sequencing, and processing

Total RNA was extracted from cryopreserved mononuclear cells (MNCs) of *EP300-ZNF384* positive B-ALL patients (*n* = 2) and healthy donors (*n* = 8) using an AllPrep DNA/RNA Mini Kit (Cat. 80,204; Qiagen, Hilden, Germany). After quality control of RNA concentration and purity, libraries were prepared according to the protocol of the TruSeq RNA Sample Preparation Kit (Illumina, San Diego, CA, USA), and library quality was assessed using a Bioanalyzer 2100 (Agilent Technologies, Santa Clara, CA, USA). Whole transcriptome sequencing was performed on a NovaSeq platform with a paired-end 150-bp read-length by the Novogene Company (Beijing, China). All sequencing data were mapped to the reference genome (hg38) using STAR (v2.7.6a) [28], and gene expression levels were measured as reads per kilobase per million mapped reads (RPKM) obtained using the Cufflinks package (v2.2.1) [29] guided by transcript coordinates according to the gene annotation format (GTF) file from GENCODE (Release 27, GRCh38).

### Public RNA-seq data analysis

To obtain the expression features of *EP300-ZNF384*-positive B-ALL patients, the expression matrix was downloaded from Therapeutically Applicable Research to Generate Effective Treatments ALL Phase II (TARGET-ALL-P2: https://ocg.cancer.gov/programs/target/), and subtype information was obtained from Gu et al. [30]. The TARGET-ALL-P2 cohort included 204 patients with B-ALL, consisting of seven *EP300-ZNF384*-positive patients, seven other *ZNF384* rearrangement patients, and 197 *EP300-ZNF384*-negative patients. We compared the expression profiles between *EP300-ZNF384*-positive and -negative patients using the “limma” package (v3.46.0) in R (http://cran.r-project.org/, v3.6), and the cutoffs for significantly differential expressed genes (DEGs) were set as fold change (FC) > 2 and *P* < 0.01. Gene set enrichment analysis (GSEA) was performed between *EP300-ZNF384*-positive and -negative patients using the Molecular Signatures Database (MSigDB; http://www.broadinstitute.org/gsea/msigdb/index.jsp). Volcano plot and heatmap were drawn using the “ggplot2” R package (v3.2.1).

### Plasmids

The *EP300-ZNF384* fusion gene and wild-type *ZNF384* were cloned from B-ALL patient samples using the Phanta Max Master Mix (Cat. P525; Vazyme, Nanjing, China) and inserted into the pLVX plasmid to construct pLVX-*EP300-ZNF384*, pLVX-*EP300-ZNF384*-3×flag, pLVX-*ZNF384*, and pLVX-*ZNF384*-3×flag respectively. For construction of the luciferase reporter vectors, a series of the sequences with different 5’ flanking regions of the *IL3RA* promoter were amplified from human genomic DNA with indicated primers and inserted into the pGL3-basic plasmid using the ClonExpress Ultra One Step Cloning Kit (Cat. C115; Vazyme). The EP300-ZNF384-binding site was mutated using the Mut Express II Fast Mutagenesis Kit V2 (Cat. C214; Vazyme) to construct mutant reporter plasmids (-234/-222 mutation and − 234/-222 deletion). Individual clones of each construct were confirmed by DNA sequencing. The primer sequences are listed in Table S1.

### ShRNA

The sequences targeting *IL3RA* (sh*IL3RA*-1: GCAGT GAACA ATAGC TATTG C; sh*IL3RA*-2: GGATT CATGA CGTGG ATTTC T; sh*IL3RA*-3: GGTGT CGTTT CGATG ACATC T; sh*IL3RA*-4: GGAAC GTACA CAGTA CAAAT A) were cloned into the pLKO lentiviral vector (Addgene, Watertown, MA, USA). An empty pLKO lentiviral vector was used as a negative control.

### Lentivirus packaging and transfection

HEK-293T cells were co-transfected with the lentiviral constructs, packaging plasmid (psPAX2) and envelope plasmid (pMD2.G) at a ratio of 4:3:1 using jetPRIME® (Polyplus, Illkirch, France). The viruses were collected, concentrated, and added to the cells using polybrene (Cat. HY-112,735; MedChemExpress, Monmouth Junction, NJ, USA) for 24 h. The infected cells were further selected with 2 µg/mL puromycin (Cat. S7417; Selleck) or 400 µg/mL hygromycin B (Cat. ST1389; Beyotime, Shanghai, China), to obtain stably transfected cells.

### Flow cytometry

The cells were washed with a staining solution (2% FBS in PBS) and resuspended in a staining solution containing fluorochrome-conjugated antibodies for 30 min. Propidium iodide (PI) was added to exclude dead cells from analysis. After staining, the cells were washed and analyzed using a Cytoflex flow cytometer (Beckman Coulter, Brea, CA, USA). Analysis was performed using CytExpert and FlowJo software. For xenograft sample analysis, BM cells were flushed from the femurs of mice and subjected to red blood cell lysis (Cat. R1010; Solarbio, Beijing, China) according to the manufacturer’s protocol. BM cells were then strained through a 70-µm mesh cell strainer and immunophenotyped using standard methodology.

### Western blot analysis

Fresh cells were lysed on ice in radioimmunoprecipitation assay buffer (50 mM Tris, pH 7.4, 150 mM NaCl, 1% NP-40, 0.5% sodium deoxycholate, 0.1% sodium dodecyl sulfate and 1 mM phenylmethylsulfonyl fluoride) containing protease and phosphatase cocktail inhibitors (Cat. HY-K0010 and Cat. HY-K0021; MedChemExpress) for 30 min. The lysates were then cleared by centrifuging at 12,000 g for 10 min. The protein concentration was quantified using the Bradford assay. Protein lysates were separated by sodium dodecyl sulfate-polyacrylamide gel electrophoresis (SDS-PAGE), transferred to a nitrocellulose membrane (Millipore, Burlington, MA, USA), and immunoblotted with specific antibodies. Protein expression was detected using a ChemiDoc XRS Imaging System (Bio-Rad Laboratories; Hercules, CA, USA) with an enhanced chemiluminescence reagent (Advansta, San Jose, CA, USA).

### RNA extraction and RT-PCR/qPCR

Total RNA was extracted from cultured cells or patient samples using AG RNAex Pro Reagent (Cat. AG21102; Accurate Biology, Changsha, China), and RT was performed using the HiScript II Q RT SuperMix kit (Cat. R223; Vazyme) according to the manufacturer’s instructions. QPCR was performed on a Bio-RAD CFX96 fluorescence quantitative PCR instrument by using the ChamQ Universal SYBR qPCR Master Mix (Cat. Q711; Vazyme) and the relative expression of the interested genes was calculated by the 2^−ΔΔCt^ method. Human *ACTB* was used as an internal control. The primer sequences are listed in Table S2.

### Cell line-derived xenograft (CDX) models

The NOD⁃*Prkdc*^scid^*Il2rg*^null^ (NYG) mice were purchased from Liaoning Changsheng Biotechnology co., Ltd. (China). All animal studies were approved by the Institutional Animal Care and Use Committee of Dalian Medical University and carried out in accordance with established institutional guidelines and approved protocols (AEE22111). NALM-6*-EP300-ZNF384* and NALM-6-*EP300-ZNF384*/sh*IL3RA* cells (5 × 10^5^) were intravenously injected into NYG mice (5-8 weeks old). Mice were fed doxycycline (Dox, 2 mg/mL)-containing water supplemented with 3% sucrose, which was replaced every 2 d. Mice were weighed daily. Four mice were sacrificed 20 d post-transplantation to monitor engraftment. The remaining six mice were euthanized after signs of disease (10% weight loss, disruption of locomotor coordination, lack of grooming, and lethargy), and hematopoietic tissues (BM from femurs, spleen, and liver) were harvested. For the doxorubicin susceptibility analysis, NALM-6-vector, NALM-6-*EP300*-*ZNF384*, and NALM-6-*ZNF384* cells (5 × 10^5^) were intravenously injected into NYG mice. After 10 d, doxorubicin (8 mg/kg) was administered intraperitoneally. The experiments were terminated on day 17, and hematopoietic tissues (BM from the femurs and liver) were harvested.

### Hematoxylin and eosin (HE) staining and Wright–Giemsa staining

Spleen and liver tissues were fixed with 10% neutral-buffered formalin for 24 h. Tissue sections were prepared, stained with HE staining solution, and examined by a pathologist blinded to the experimental groups. Representative images were acquired at ×200 magnification using an Olympus BX63 microscope equipped with a DP73 camera (Olympus, Tokyo, Japan). BM smears were prepared from mice and stained with Wright-Giemsa stain solution (Cat. G1020; Solarbio) according to the manufacturer’s protocol. The cell morphology was observed under an Olympus BX63 microscope.

### Dual luciferase assay

HEK-293T cells were co-transfected with 200 ng of pLVX-*EP300-ZNF384*, pLVX-*ZNF384*, or the pLVX empty vector in combination with 200 ng of pGL3-*IL3RA* promoter and 10 ng pRL-TK renilla plasmid using the jetPRIME® (Polyplus). After transfection for 24 h, both firefly luciferase and *Renilla* luciferase activities were detected with an EnSpire Multimode Plate Reader (PerkinElmer, Waltham, MA, USA) using the Dual-Luciferase Reporter Assay System (Cat. E1910; Promega, Madison, WI, USA) according to the manufacturer’s instructions. Firefly luciferase activity was normalized to *Renilla* luciferase activity to control transfection efficiency. Primers used for the luciferase constructs are listed in Table S1.

### Chromatin immunoprecipitation (ChIP)

The pLVX-*EP300-ZNF384*-3×flag, pLVX-ZNF384-3×flag and pLVX-3×flag plasmids were respectively transfected into HEK-293T cells for 48 h using jetPRIME® (Polyplus). ChIP assay was conducted using the ChIP-IT ® Express kit (Cat. 53009; Active Motif, Carlsbad, CA, USA), according to the manufacturer’s specifications. The cells were cross-linked with 1% formaldehyde (Cat. 28908; Thermo Fisher Scientific) and lysed with lysis buffer. Cross-linked chromatin was sheared into fragments with an average size of 300-1500 bp and immunoprecipitated using antibodies against flag and IgG (Cat. A7028; Beyotime). Specific PCR amplification was conducted using primers compatible with *IL3RA* or *MMP7* promoter sequences. The primer sequences are listed in Table S3.

### ELISA

CD19^+^ cells were infected with lentiviruses overexpressing the empty vector, *EP300-ZNF384*, or *ZNF384* in serum-free medium. Cell culture media were collected at 48 h and the secretion of IL-3 was determined using an ELISA kit for IL-3 (Cat. SEA076Hu; Cloud-Clone Corp.) according to the manufacturer’s instructions.

### Cell viability assay

Cell viability was determined using the CCK-8 assay. Briefly, NALM-6 cells harboring the empty vector, *EP300-ZNF384*, or *ZNF384* were seeded into a 96-well plate at a density of approximately 2 × 10^4^ cells per well and treated with the indicated drug concentrations for 48 h. At the end of the experiment, 10 µL of CCK-8 reagent (Cat. MA0218; Meilunbio) was added to each well and incubated at 37 °C for 2 h. Optical densities at 450 nm were determined using a Multiscan Spectrum SpectraMax M2 (Molecular Devices, San Jose, CA, USA). The relative viability values of cells were calculated.

### Methylcellulose-based colony forming cell (CFC) assay

Single-cell suspensions were plated in 96-well plates (Corning) at a density of 200 cells/well and cultured in IMDM medium supplemented with 10% FBS (Gibco) and 1.27% methylcellulose (Sigma). The cells were treated as indicated. After 10 d, the colonies were photographed and counted under an inverted microscope (Olympus).

### Apoptosis analysis

An Annexin V-FITC/PI Apoptosis Kit (Cat. E-CK-A211; Elabscience) was used to detect the apoptosis-inducing effects of doxorubicin in NALM-6 cells. The cells were resuspended in a 500 µL 1× binding buffer with 5 µL Annexin V-FITC and 5 µL PI and incubated at room temperature for 15 min in dark. Apoptosis was detected using flow cytometer (CytoFLEX, Beckman Coulter). The results were analyzed using FlowJo software.

### Statistical analysis

Statistical analyses were performed using the GraphPad Prism 8 software (GraphPad Software Inc., La Jolla, CA, USA). The unpaired Student’s *t*-test was used to perform statistical analyses between two groups. One-way analysis ANOVA was used for comparisons between two groups in multiple groups. Survival data were analyzed using the log-rank test, and survival curves were assessed using the Kaplan-Meier method. The levels of significance were set at *P < 0.05, **P < 0.01, and ***P < 0.001. The data generated and/or analyzed in the current study are available from the corresponding author upon reasonable request.

## Results

### EP300-ZNF384 promotes the expression of *IL3RA* in leukemia cells

**Fig. 1:**
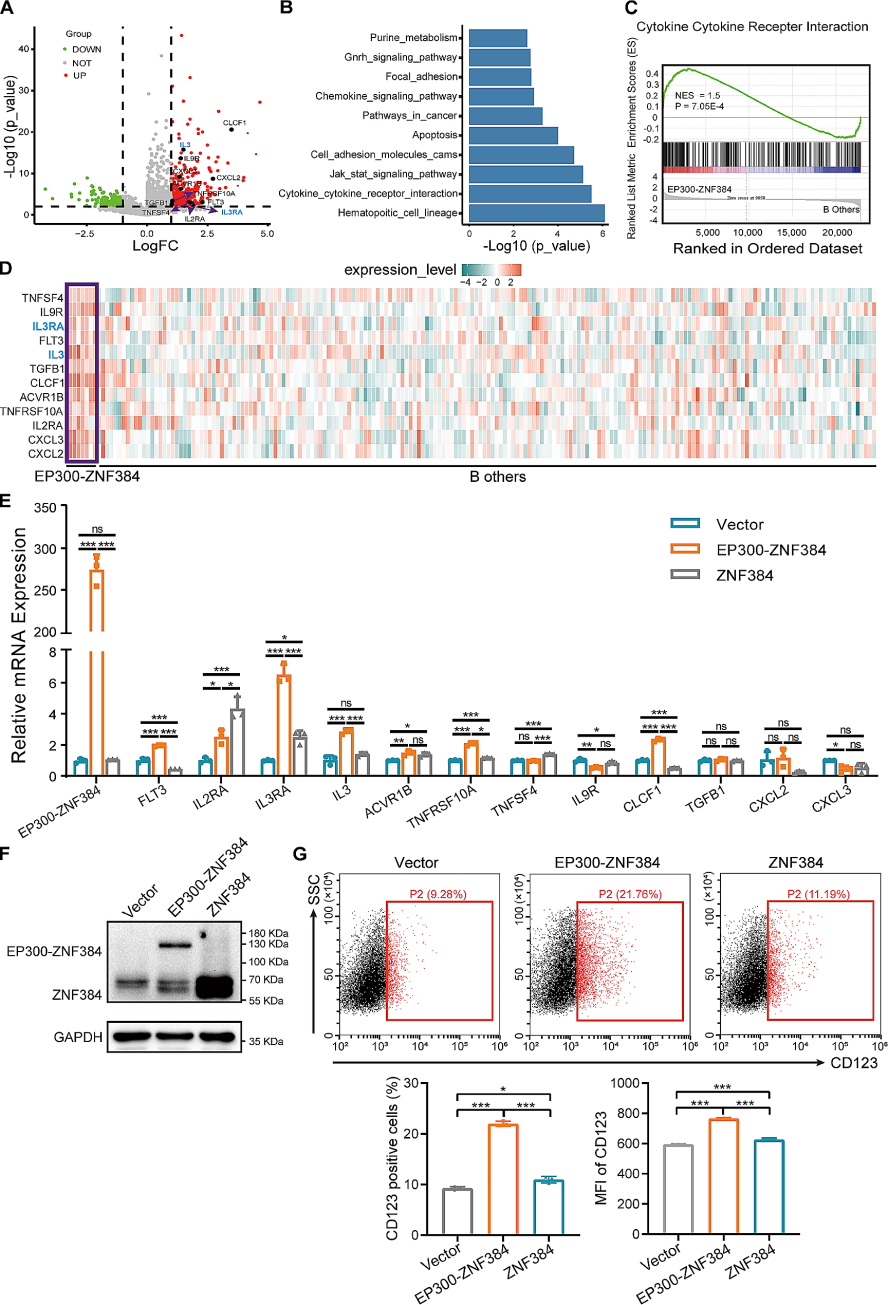
*EP300-ZNF384* promotes the expression of *IL3RA* in B-cell acute lymphoblastic leukemia (B-ALL). **(A)** Volcano plot of comparative analysis between *EP300-ZNF384* positive (*n* = 7) and negative (*n* = 190) patients in the TARGET-ALL-P2 cohort. Upregulated (*n* = 346) and downregulated (*n* = 216) genes are colored by red and green (fold change > 2 and *P* < 0.01). The labeled texts showed the upregulated genes in the cytokine-cytokine receptor interaction pathway. **(B)** Bar plot of enriched Kyoto Encyclopedia of Genes and Genomes gene sets using the differentially expressed genes (fold change > 2 and *P* < 0.01) from the comparison of the *EP300-ZNF384* positive and negative patients in the TARGET-ALL-P2 cohort. **(C)** Representative GSEA plot of *EP300-ZNF384*-positive patients compared with *EP300-ZNF384*-negative patients. The normalized enrichment score (NES) and nominal P-values are shown in the graph. **(D)** Heatmap of 12 upregulated genes in the cytokine-cytokine receptor interaction pathway in *EP300-ZNF384*-positive patients. *IL3* and *IL3RA* are labeled with blue. **(E)** The empty vector, *EP300-ZNF384*, and *ZNF384* were overexpressed in NALM-6 cells through lentivirus-mediated gene transfer. Puromycin (2 µg/mL)-selected cells were collected for RT-qPCR. The expression of *EP300-ZNF384* together with those of 12 upregulated genes in the cytokine and cytokine receptor interaction pathway were determined relative to *ACTB* expression. Data represent the mean ± SD. *P < 0.05, **P < 0.01, ***P < 0.001. **(F)** Immunoblotting analysis of the EP300-ZNF384 fusion and wild-type ZNF384 proteins in the empty vector, *EP300-ZNF384*, and *ZNF384*-overexpressing NALM-6 cells. **(G)** Surface expression of CD123 was evaluated by flow cytometry on NALM-6 cells with the empty vector, *EP300-ZNF384* fusion gene, and *ZNF384*. The proportion of CD123-positive cells and the median fluorescence intensity of CD123 were quantified. *P < 0.05, ***P < 0.001

To fully understand the oncogenic mechanism underlying *EP300-ZNF384*, we compared the expression profiles of *EP300-ZNF384* fusion-positive B-ALL patients to negative patients using publicly available data from TARGET (*n* = 204). The volcano plot displays differentially expressed genes (DEGs), including 346 upregulated and 216 downregulated genes in *EP300-ZNF384*-positive patients (Fig. 1A). Functional annotation analysis of DEGs revealed that the top three enriched Kyoto Encyclopedia of Genes and Genomes pathways were hematopoietic cell lineage, cytokine-cytokine receptor interaction, and the JAK/STAT signaling pathway (Fig. 1B). Using GSEA, we identified that the cytokine-cytokine receptor interaction pathway was significantly upregulated in the *EP300-ZNF384-*positve patient samples (Fig. 1C). Furthermore, we found that 12 cytokine-cytokine receptor interaction pathway genes were significantly upregulated in *EP300-ZNF384*-positive patients (Fig. 1A and D). These data indicate that the *EP300-ZNF384* fusion may promote the expression of key cytokines or cytokine receptors. In particular, both *IL3* and *IL3RA* were significantly upregulated (Fig. 1A and D), suggesting that IL3RA signaling may be a downstream effector of the EP300-ZNF384 fusion protein.

To confirm the specific cytokine signaling pathway involved in *EP300-ZNF384*-induced leukemogenesis, the *EP300-ZNF384* fusion gene, wild-type *ZNF384* (ZNF384), and empty vector (vector) were expressed in NALM-6 cells. The expression of *EP300-ZNF384* was confirmed by RT-qPCR (Fig. 1E) and the 130 kDa EP300-ZNF384 fusion protein was detected by immunoblotting for ZNF384 (Fig. 1F). RT-qPCR was performed to evaluate the expression of the 12 upregulated genes within the cytokine-cytokine receptor interaction pathway. Overexpression of *EP300-ZNF384* resulted in significant upregulation of *FLT3, IL3RA, IL3, ACVR1B, TNFRSF10A*, and *CLCF1* and significant downregulation of *IL9R* and *CXCL3* in NALM-6 cells (*P* < 0.05, Fig. 1E). Among the six upregulated genes, *FLT3* and *CLCF1* have been previously reported to be upregulated in *EP300-ZNF384*-positive cells (*P* < 0.001). Notably, the overexpression of *EP300-ZNF384* led to the most significantly upregulated expression of *IL3RA* (∼ 6.5-fold, *P* < 0.0001) in NALM-6 cells (Fig. 1E). Similar results were also observed in BALL-1, Daudi, and KG-1α cells with *EP300-ZNF384*, supporting that EP300-ZNF384 fusion protein promotes the expression of *IL3RA* in both B and myeloid leukemia cell lines (Fig. S1A-C). Further, we detected the expression of IL3Rα (CD123) on the cell membrane by flow cytometry. We detected a basal expression of CD123 (9.28%) in vector expressing NALM-6 cells (Fig. 1G). In response to *EP300-ZNF384* expression, we observed a 2.3-fold increase in the CD123^+^ subpopulation and a 1.29-fold increase in CD123 fluorescence intensity in NALM6 cells (Fig. 1G). A similar observation was made for BALL-1 cells (Fig. S1D). Moreover, we observed that *EP300-ZNF384* promotes the expression of *IL3* in NALM-6 cells (∼ 2.6-fold, *p* < 0.0001, Fig. 1E), but not in BALL-1, Daudi, and KG-1α cells. These results demonstrate that EP300-ZNF384 promoted the expression of *IL3RA* in a wide range of leukemia cells.

### Increased expression of *IL3RA* by *EP300-ZNF384* promotes B-ALL cell proliferation with or without IL-3

**Fig. 2:**
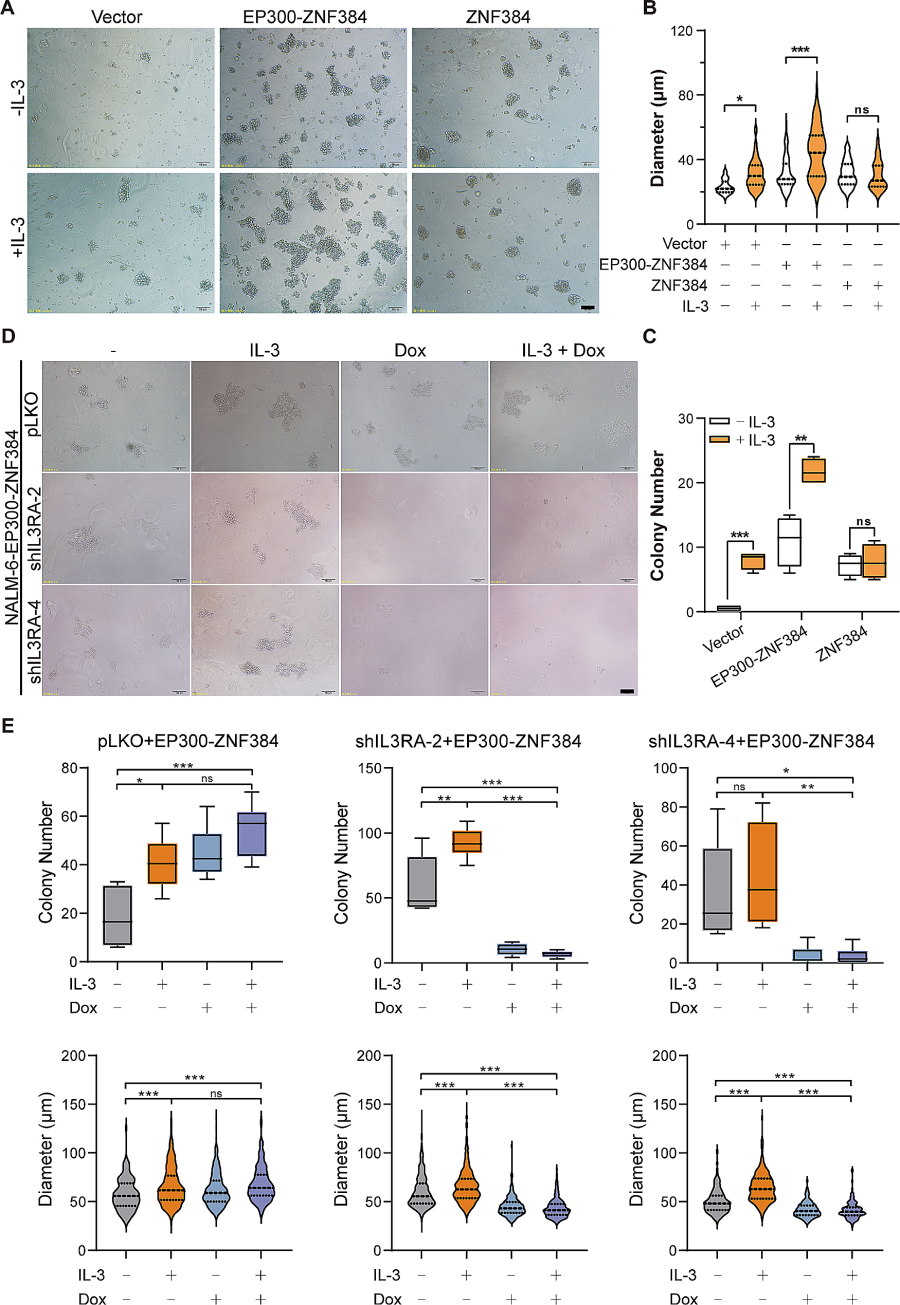
Increased expression of *IL3RA* by *EP300-ZNF384* promotes B-cell acute lymphoblastic leukemia (B-ALL) cell proliferation with or without IL-3. **(A)** NALM-6 cells overexpressing *EP300-ZNF384*, *ZNF384*, and the empty vector were subjected to a colony forming cell (CFC) assay in the presence or absence of IL-3 (10 ng/mL). Scale bar: 50 μm. Data derived from A was used for quantitative analysis of colony size **(B)** and colony number **(C)**. *P < 0.05, **P < 0.01, ***P < 0.001, ns, no significance. **(D)** The NALM-6 cells were co-transfected with the shRNA vector (sh*IL3RA* or empty vector) and overexpression vector (*EP300-ZNF384* or empty vector) via lentivirus-mediated gene transfer. Hygromycin B (400 µg/mL) and puromycin (2 µg/mL) double selected cells were subjected to CFC assay. Scale bar: 100 μm. **(E)** Data derived from D were used for quantitative analysis of colony size (diameter) and number. *P < 0.05, **P < 0.01, ***P < 0.001, ns, no significance

IL3Rα functions as a specific receptor of the IL-3 ligand, which is necessary for IL-3 activated cell proliferation [24]. To investigate whether increased expression of *IL3RA* was responsible for IL-3 induced cell proliferation of *EP300-ZNF384*-positive cells, we performed a CFC assay. In the absence of IL-3, both *EP300*-*ZNF384* and *ZNF384* enhanced the colony forming ability of NALM-6 cells, as evidenced by elevated colony number (average colony number: Vector, 0.5, *EP300-ZNF384*, 11, *ZNF384*, 7.25) and size (average colony size: Vector, 23.13 μm, *EP300-ZNF384*, 31.2 μm, *ZNF384*, 30.99 μm) (Fig. 2A-C). Notably, EP300-ZNF384-expressing NALM-6 cells displayed stronger colony-forming ability than ZNF384-expressing NALM-6 cells, as evidenced by significantly increased colony number (*P* < 0.0001). In the presence of IL-3, the colony formation was significantly elevated in both *EP300-ZNF384*- and vector-expressing NALM-6 cells (average colony number: Vector, 8, *EP300-ZNF384*, 21.75, *ZNF384*, 7.75; average colony size: Vector, 30.94 μm, *EP300-ZNF384*, 43.78 μm, *ZNF384*, 30.09 μm). NALM-6 cells harboring *EP300-ZNF384* showed the strongest colony-forming ability in response to IL-3 treatment (Fig. 2B-C). Western blotting confirmed that IL-3 specifically increased the phosphorylation of STAT5 in *EP300-ZNF384*-positive NALM-6 cells (Fig. S2A). These data indicate that *EP300-ZNF384* induced IL3RA elevation to make it sensitive to IL-3 stimulation, resulting in augmented activation of STAT5 and enhanced colony formation in B-ALL cells.

To understand the functional involvement of *IL3RA* in *EP300-ZNF384*-positive B-ALL cells, we used a doxycycline-inducible shRNA expression system to knock down *IL3RA* (sh*IL3RA*-1, sh*IL3RA*-2, sh*IL3RA*-3, and sh*IL3RA*-4) in NALM-6 cells. An empty vector served as a control. The knockdown efficiency of *IL3RA* was assessed by RT-qPCR. Doxycycline supplementation significantly reduced the expression of *IL3RA* in both sh*IL3RA*-2- and sh*IL3RA*-4-expressing NALM-6 cells (Fig. S2B). Then, an *EP300-ZNF384*-expressing vector as well as control vector was transfected into sh*IL3RA-*expressing NALM-6 cells. We performed a CFC assay to evaluate the proliferative ability of *EP300-ZNF384*- or vector-expressing NALM-6 cells with or without *IL3RA*. Due to the basal expression of CD123, IL-3 modestly promote the proliferation of vector expressing NALM-6 cells (Fig. S2C). The knockdown of *IL3RA* (sh*IL3RA*-2 and sh*IL3RA*-4) by doxycycline only induced a modest reduction in colony formation in empty vector expressing-NALM-6 cells, with or without IL-3 (middle and right panels of Fig. S2C). In pLKO and *EP300-ZNF384*-expressing NALM-6 cells, IL-3 significantly upregulated colony formation (left panel of Fig. 2E). Consistent with this, interference with *IL3RA* completely abolished the colony formation ability of *EP300-ZNF384*-expressing NALM-6 cells with or without IL-3 (middle and right panels of Fig. 2E). In sh*IL3RA*-4-expressing NALM-6 cells, we confirmed that doxycycline supplementation efficiently reduced the expression of *IL3RA* induced by *EP300-ZNF384* (Fig. S2D). Furthermore, silencing of *IL3RA* abrogated the IL-3-induced phosphorylation of STAT5 at Y694 in *EP300-ZNF384* expressing NALM-6 cells (Fig. S2E). These results collectively indicate that *EP300-ZNF384*-expressing cells acquired a growth advantage by upregulating the expression of *IL3RA*.

### Conditional knockdown of *IL3RA* impairs the expansion of *EP300-ZNF384* positive B-ALL cells in mice

**Fig. 3:**
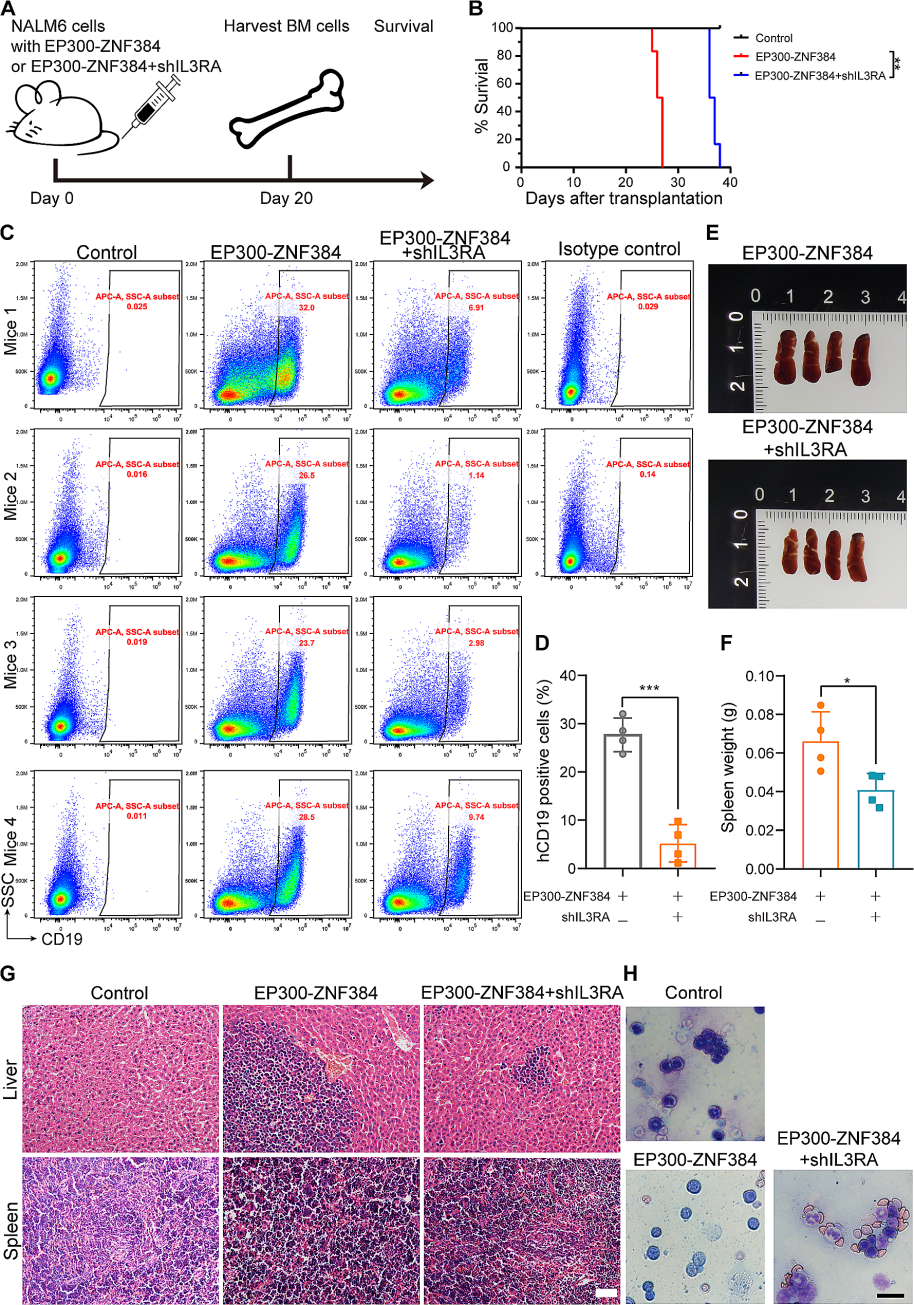
Conditional knockdown of *IL3RA* impairs the engraftment of *EP300-ZNF384*-positive B-cell acute lymphoblastic leukemia (B-ALL) cells in mice. **(A)** The schematic diagram of the in vivo experiment. NALM-6 cells expressing *EP300-ZNF384* or *EP300-ZNF384* and sh*IL3RA* were transplanted into immune-deficient NOD⁃*Prkdc*^scid^*Il2rg*^null^ mice. Mice were then treated with doxycycline (Dox, 2 mg/mL) in drinking water. After 20 d, four mice were sacrificed and femur bone marrow cells were collected for leukemia cell analysis. The remaining six mice were euthanized upon signs of disease. **(B)** Kaplan–Meier survival curve of mice injected with *EP300-ZNF384* or *EP300-ZNF384* and sh*IL3RA*-expressing NALM-6 cells. **P < 0.01 **(C)** hCD19 fractions within the femur bone marrow of each mouse. **(D)** Column plots summarizing data from all animals analyzed as in C. ***P < 0.001. **(E)** Changes in the spleen in *EP300-ZNF384*-expressing NALM-6-transplantable mice in response to *IL3RA* knockdown. **(F)** Spleen weights are presented by column plots. ***P < 0.001. **(G)** The leukemic invasions in spleen and liver were analyzed by hematoxylin and eosin staining. Scale bar: 50 μm. **(H)** Wright–Giemsa staining of BM cells from mice with NALM-6 cells expressing *EP300-ZNF384* or *EP300-ZNF384* and sh*IL3RA*. Scale bar: 20 μm

To further define the oncogenic role of *IL3RA* in *EP300-ZNF384*-expressing B-ALL cells in vivo, *EP300-ZNF384*-positive NALM-6 cells expressing sh*IL3RA*-4 or a control vector were transplanted into immunodeficient NYG mice (Fig. 3A). Mice that received *EP300-ZNF384*-expressing NALM-6 cells rapidly developed leukemia, with a median survival time of 26 d. However, *IL3RA* depletion remarkably extended the survival of recipient mice, with a median survival of 36 d (*n* = 6, *P* < 0.01; Fig. 3B). Four mice were sacrificed at 20 d after transplantation, and hematopoietic tissues, such as BM from the femurs, spleen, and liver were harvested for leukemia expansion and infiltration analysis. *EP300-ZNF384* positive NALM-6 cells harboring sh*IL3*RA exhibited a much lower leukemia burden in the mouse BM than cells harboring the empty vector *EP300-ZNF384* (*n* = 4, *P* = 0.0001, Fig. 3C-D). Consistently, our results showed that *IL3RA* depletion markedly reduced the infiltration of *EP300-ZNF384*-expressing NALM-6 cells into the spleen and liver (Fig. 3E-G). Morphological analysis revealed excessive numbers of immature blasts in the BM smears of mice that received *EP300-ZNF384*-expressing NALM-6 cells. In the sh*IL3*RA group, both mature myeloid cells and immature blasts were observed, suggesting that *IL3RA* depletion impaired the expansion of *EP300-ZNF384*-expressing NALM-6 cells in the BM and the infiltration in liver and spleen. Collectively, these results indicate that the expression of *IL3RA* is crucial for the propagation of *EP300-ZNF384*-expressing NALM-6 cells in vivo.

### EP300-ZNF384 fusion protein transactivates the promoter activity of *IL3RA*

**Fig. 4:**
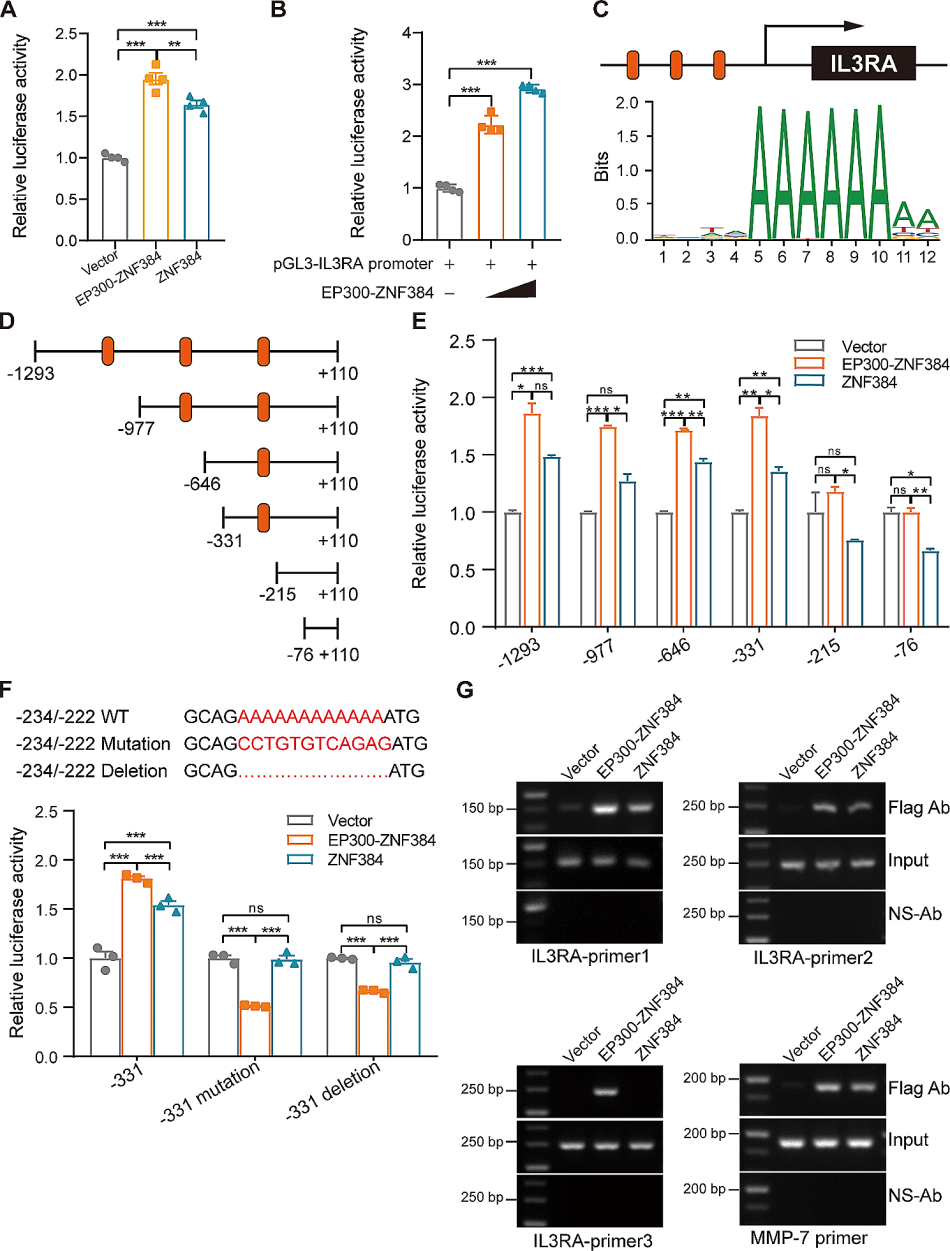
EP300-ZNF384 fusion protein transactivates the promoter activity of *IL3RA* **(A)** *EP300-ZNF384*- or *ZNF384*-expressing vectors were co-transfected with the PGL3-*IL3RA*-promoter and pRL-*TK* into HEK-293T cells. After 24 h, dual luciferase assay was performed. Data represents the mean ± SD. **P < 0.01, ***P < 0.001. **(B)** Increasing doses of *EP300-ZNF384*-expressing vectors and the luciferase reporter vectors were co-transfected into HEK-293T cells. Luciferase analysis for the *IL3RA* promoter was performed. ***P < 0.001. **(C)** Diagram showing the putative binding sites of ZNF384 in *IL3RA* promoter (orange). **(D)** Truncated mutations of the *IL3RA* promoter are shown. Numbering is indicated with respect to the transcriptional start site. **(E)** Luciferase analysis for the truncated *IL3RA* promoters was performed. *P < 0.05, **P < 0.01, ***P < 0.001, ns, no significance. **(F)** Schematic presentation of the *IL3RA* promoter mutations used in this study. The mutated *IL3RA* promoter reporter along with pRL-*TK* were co-transfected with the empty vector, *EP300-ZNG384*, or *ZNF384*-expressing vectors into HEK-293T cells for 24 h. Dual-luciferase assay was performed. ***P < 0.001, ns, no significance. **(G)** HEK-293T cells were transfected with flag-tagged *EP300-ZNF384*- and *ZNF384*- expressing vectors for 48 h. Chromatin fragments of these cells were immunoprecipitated with a flag or nonspecific antibody (ns-Ab). PCR was conducted with primers corresponding to the genomic *IL3RA* and *MMP7* promoter sequences. *MMP7* served as a positive control

ZNF384 is a C2H2-type zinc-finger transcription factor. Thus, there is reason to believe that EP300-ZNF384 may target sites carrying the ZNF384 DNA-binding motif. To determine whether EP300-ZNF384 regulates *IL3RA* gene expression at transcriptional level, the promoter of *IL3RA (*from − 1293 to + 110) was cloned into the pGL3 reporter construct. Dual-luciferase reporter constructs were co-transfected with *EP300-ZNF384, ZNF384*, or an empty vector into HEK-293T cells. We found that the luciferase activity was increased in HEK-293T cells bearing *EP300-ZNF384* and *ZNF384* compared to the empty vector (Fig. 4A). The EP300-ZNF384 fusion displayed stronger transcriptional activity of *IL3RA* than wild-type ZNF384, indicating that the fusion of *EP300* to *ZNF384* enhanced the transcriptional activity of *ZNF384* on *IL3RA* promoter. We found that EP300-ZNF384 activated *IL3RA* transcription in a dose-dependent manner, further demonstrating that the EP300-ZNF384 fusion protein transactivated the promoter activity of *IL3RA* (Fig. 4B).

To determine the region of *IL3RA* promoter regulated by the EP300-ZNF384 fusion, we performed motif prediction using the JASPAR database and found that EP300-ZNF384 could potentially bind to three regions; namely -234 bp/-223 bp, -735 bp/-731 bp, and -1057 bp/-1035 bp; in the human *IL3RA* promoter (Fig. 4C). Dual luciferase analysis showed that EP300-ZNF384 failed to activate the transcription at the *IL3RA* promoter when the − 331 bp/-215 bp region of *IL3RA* promoter was deleted (Fig. 4D, E). To identify the critical nucleotides within this region, deleted or substituted mutations of the -234 bp/-222 bp were introduced into the luciferase reporter vector (the upper panel of Fig. 4F). Both deletion of the -234 bp/-222 bp and replacement of the sequence with CCTGTGTCAGAG shifted the promoter from active to inactive status (the lower panel of Fig. 4F). The results of ChIP assay further indicated that EP300-ZNF384 interacted with the -234 bp/-222 bp region of the *IL3RA* promoter (Fig. 4G). These data collectively demonstrate that the EP300-ZNF384 fusion protein transactivates the promoter activity of *IL3RA* by binding to the − 234 bp/-222 bp region.

### *EP300-ZNF384* elevates the expression of IL3Rα (CD123) in B precursor cells from healthy individuals

**Fig. 5:**
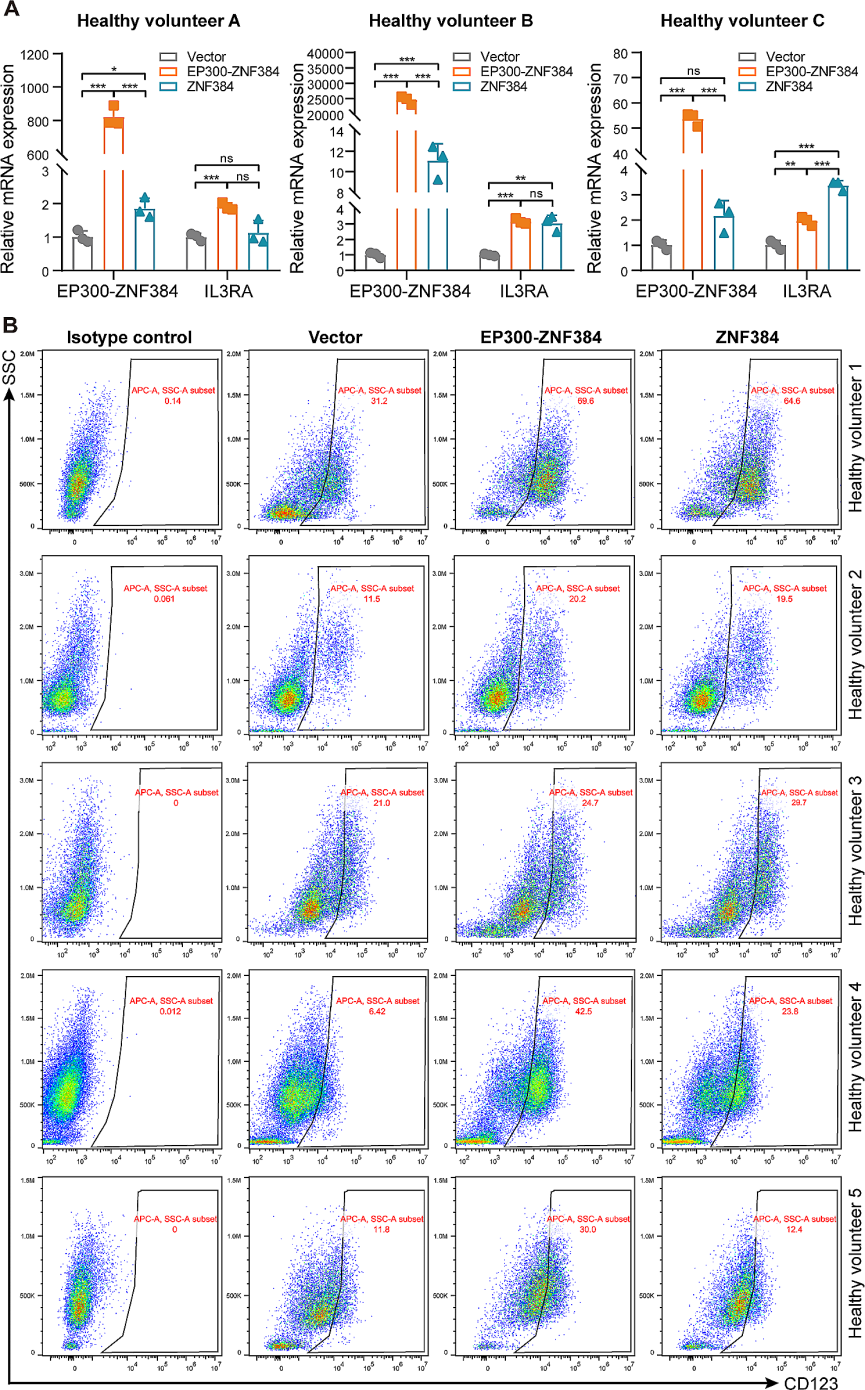
*EP300-ZNF384* elevates the expression of CD123 in B precursor cells. **(A)** CD19-positive cells were enriched with magnetic beads from peripheral blood mononuclear cells of healthy volunteers. Empty vector, *EP300-ZNF384-*, and *ZNF384-*expressing vectors were transfected into CD19-positive cells by lentivirus-mediated gene transfer. After 48 h, cells were collected for reverse-transcription quantitative PCR assay to assess the transcription of *IL3RA*. Data are represented as mean ± SD. *P < 0.05, **P < 0.01, ***P < 0.001, ns, no significance. **(B)** Representative flow cytometry dot plots illustrating CD123 fractions in CD19 positive B precursor cells in response to *EP300-ZNF384* or *ZNF384* expression

To fully understand whether *EP300-ZNF384* promotes the expression of *IL3RA* in the initial stages of leukemogenesis, hCD19^+^ B precursor cells were isolated from healthy individual-derived PBMCs and transfected with lentiviruses expressing *EP300-ZNF384*, *ZNF384*, or the vector. Compared with the empty vector, *EP300-ZNF384* strikingly promoted the expression of *IL3RA* in hCD19^+^ cells derived from three healthy individuals (Fig. 5A). The CD123 expression was then determined by flow cytometry. Less than 1% of the samples was stained with negative isotype controls, which was set as the gating region. In comparison with the empty vector, *EP300-ZNF384* elevated the expression of CD123 on cell surface in hCD19^+^ cells derived from 5 healthy individuals (Fig. 5B and Fig. S3A-B).

Our previous RNA sequencing analysis demonstrated that the expression of *IL3* was significantly upregulated in B-ALL patients with *EP300-ZNF384* fusion lesions compared to that in B-ALL patients without *EP300-ZNF384* fusion lesions (Fig. 1D). We demonstrated that *EP300-ZNF384* induced the expression of *IL3* in NALM-6 cells. However, the expression of *IL3* is poorly detected by RT-qPCR in other leukemia cell lines. We then determined expression patterns during hematopoiesis using the BloodSpot website (https://servers.binf.ku.dk/bloodspot/). In the normal human hematopoiesis (DMAP) database, *IL3* was mildly expressed in early- and pro-B cells (Fig. S3C). Furthermore, we performed RT-qPCR and ELISA assays to investigate whether *EP300-ZNF384* induced the expression of *IL3* in CD19^+^ B precursor cells. Our data demonstrate that both the expression and secretion of IL3 were promoted by *EP300-ZNF384* in B precursor cells (Fig. S3D, E). Our results demonstrated that *EP300-ZNF384* promotes B-ALL cell expansion by activating the expression of IL3Rα and the secretion of IL-3 at the initial stage of leukemogenesis. Further, this implies that *EP300-ZNF384* boosts IL3RA autocrine signaling to confer a growth advantage to B-ALL cells.

### *EP300-ZNF384* positive B-ALL cells are more sensitive to doxorubicin in vitro and in vivo

**Fig. 6:**
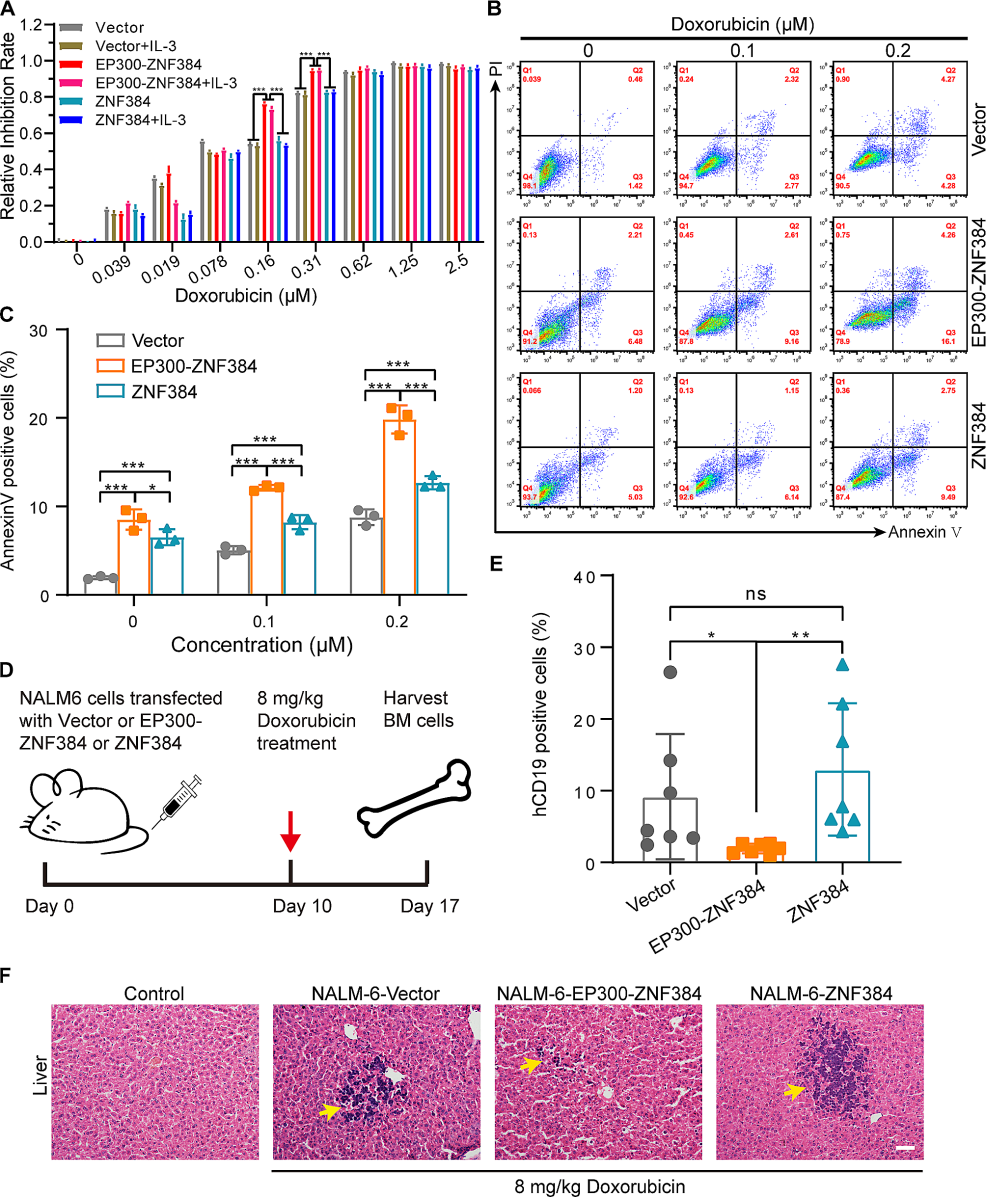
*EP300-ZNF384* positive B-cell acute lymphoblastic leukemia (B-ALL) cells are more sensitive to doxorubicin in vitro and in vivo. **(A)** Empty vector-, *EP300-ZNF384-*, and *ZNF384*-expressing NALM-6 cells were treated with increasing doses of doxorubicin for 48 h in the presence or absence of IL-3. Cell viability was determined by CCK-8 assay. Data are represented as mean ± SD. ***P < 0.001. **(B)** Empty vector-, *EP300-ZNF384-*, and *ZNF384*-expressing NALM-6 cells were treated with indicated doses of doxorubicin for 48 h. Cell apoptosis was detected by dual Annexin V-FITC/PI staining. Representative flow cytometry dot plots are presented **(C)** Column plots represents the proportion of Annexin V-positive cells. Data were represented as mean ± SD (*n* = 3). Unpaired *t*-test. *P < 0.05, ***P < 0.001. **(D)** Schematic describing generation of CDX mouse model by engrafting NOD⁃*Prkdc*^scid^*Il2rg*^null^ (NYG) mice with NALM-6 cells harboring the empty vector, *EP300-ZNF384*, or *ZNF384*, followed directly by in vivo treatment with doxorubicin (8 mg/kg, intraperitoneally). (E) Percentage of human CD19^+^ subpopulations in the bone marrow of NYG mice engrafted with empty vector or *EP300-ZNF384* or *ZNF384* expressing NALM-6 cells, followed by in vivo doxorubicin treatment. (F) Leukemia involvement in the liver was determined by H&E staining. Scale bar indicates 50 μm

A previous clinical report showed that approximately 87.5% of patients who received VDCLP or a VDCLP-like regimen of induction chemotherapy achieved complete remission [31]. We speculate that chemotherapeutic agents within the VDCLP regimen (vincristine, doxorubicin, L-asparaginase, and dexamethasone were used in this research) might sensitize B-ALL leukemia cells with *EP300-ZNF384*. We then performed the CCK-8 assay to assess the sensitivity of NALM-6 cells bearing the *EP300-ZNF384* fusion, wild-type *ZNF384*, or Vector to chemotherapeutic agents. Our results revealed that both doxorubicin and pegaspargase preferentially inhibited the cell viability of *EP300-ZNF384* expressing-NALM-6 cells in a dose-dependent manner (Fig. S4A-D). Of note, doxorubicin reduced the viability of *EP300-ZNF384*-expressing NALM-6 cells by about 10% over a wide range of concentrations (0.078–0.31 µM), as compared with cells with the empty vector (Fig. S4A). Furthermore, *EP300-ZNF384*-expressing NALM-6 cells were selectively sensitive to doxorubicin in the presence or absence of IL-3 (Fig. 6A). Apoptosis was determined by dual staining with FITC-labeled Annexin V and PI. Our results demonstrated that doxorubicin significantly induced the apoptosis of *EP300-ZNF384* expressing*-*NALM-6 cells, as evidenced by the elevated Annexin V^+^ proportion (Fig. 6B, C). Next, we examined the in vivo therapeutic potential of doxorubicin by treating NYG mice transplanted with NALM-6 cells expressing the *EP300-ZNF384*, *ZNF384*, or empty vectors (Fig. 6D). In line with our ex vivo observations, *EP300-ZNF384*-expressing NALM-6 cells responded remarkably to doxorubicin treatment in vivo, resulting in a significantly decreased percentage of hCD19^+^ cells in mouse BM and a reduced leukemia burden in the liver (Fig. 6E, F, and S5). These findings support the therapeutic value of doxorubicin in targeting *EP300-ZNF384* positive B-ALL cells.

## Discussion

The *EP300-ZNF384* fusion gene is one of the most common genetic lesions within B-ALL patients and induces leukemogenesis in murine transplantation models [9, 10]. Here, we demonstrated that the EP300-ZNF384 fusion protein substantially induces the expression of IL3Rα, which contributes to an acquired growth advantage of B-ALL cells in the presence or absence of IL-3. Mechanistically, the EP300-ZNF384 fusion protein transactivates the promoter activity of *IL3RA* by binding to an A-rich sequence localized at -222/-234 of the *IL3RA* gene. We further demonstrated that doxorubicin treatment sensitized *EP300-ZNF384* expressing B-ALL cells.

IL3Rα (also known as CD123) regulates the proliferation, survival, and differentiation of hematopoietic cells upon binding with IL-3. IL3Rα is highly expressed in multiple types of leukemia such as AML and ALL and has been recognized as a specific marker for leukemic stem cells in AML patients [20–23, 32]. The elevated expression of IL-3Rα is associated with enhanced blast proliferation and a poor prognostic phenotype in patients with AML [24–26]. Our current work disclosed that *EP300-ZNF384* increased the expression of IL3Rα, which resulted in an enhanced proliferative activity of B-ALL cells. IL-3 is a tightly regulated pleiotropic cytokine produced mainly by activated T lymphocytes [33]. Numerous studies demonstrated that IL-3 promotes proliferation and colony formation of primary leukemic cells in vitro, supporting that aberrant IL-3 signal plays significant roles in the process of leukemogenesis [34, 35]. In addition, we showed that *EP300-ZNF384* promoted the expression and production of IL-3 in B precursor cells, indicating that both autocrine and paracrine IL-3 may be related to the enhanced growth advantage of *EP300-ZNF384*-positive B-ALL cells. Collectively, our findings suggest the oncogenic mechanism underlying *EP300-ZNF384* is involved in aberrant IL3Rα signaling.

The expression of *IL3RA* is regulated by transcription factors and epigenetic regulators [36]. A recent study revealed that the *NUP98-NSD1* fusion gene induces CD123 expression in 32D cells [37]. Clinical data showed that patients with *ZNF384* rearrangements have significantly higher CD123 expression than those with non-*ZNF384* rearrangements (*BCR-ABL*, *KMT2A* rearrangement, and other B-ALL) [27]. To date, there has been no evidence that oncogenic fusion proteins directly regulate the expression of *IL3RA*. In the present study, we demonstrated that *EP300-ZNF38*4 transcriptionally promotes the expression of *IL3RA*, which enhanced the proliferative activity of B-ALL cells. We identified a EP300-ZNF384 fusion protein binding sequence at the *IL3RA* promoter and proved the fusion of *EP300* with *ZNF384* increased the transaction activity at the *IL3RA* promoter, leading to a much higher expression of IL3Rα on B-ALL cell membrane. Our findings suggest that CD123 is a direct downstream target of the EP300-ZNF384 fusion protein and could serve as a valuable marker of *EP300-ZNF384* fusion. Furthermore, our data confirmed that the previously proposed FCM scoring system, including CD10, CD13, CD33, and CD123, is reliable for distinguishing *ZNF384* rearrangements from non-*ZNF384* rearrangements with high sensitivity and specificity.

Targeting CD123 via CAR-engineered T cells, neutralizing antibodies, and IL3RA antibodies coupled to toxins has been shown to have beneficial effects on the survival of AML models [38–41]. The CD123-targeted antibody-drug conjugate demonstrated promising selective activity in preclinical models of B-ALL [42]. Our recent study showed that depletion of IL3RA completely abolished the growth advantage conferred by *EP300-ZNF384* in B-ALL cells and impaired the engraftment of *EP300-ZNF384*-positive B-ALL cells in mice. Although current work did not discuss the anti-leukemic activity of CD123-targeted therapy, given the specific expression pattern of CD123 in tumors with *EP300-ZNF384* rearrangement, our results support CD123 as a promising therapeutic target for patients with *EP300-ZNF384* fusion, suggesting that IL3Rα-targeting therapeutics may be effective for patients with EP300-*ZNF384* rearrangement. Studies on the anti-leukemia activity of CD123-targeted therapeutics are urgently needed in the next few years.

ALL treatment has mainly been based on chemotherapy and hematopoietic stem cell transplantation (HSCT) in high risk [43]. B-ALL patients with *EP300-ZNF384* have been found to achieve a higher remission rate than those without it [5, 7]. Thus, we speculated that certain agents within the chemotherapy regimens may sensitize *EP300-ZNF384*-positive leukemia cells. We performed drug screening using vincristine, doxorubicin, L-asparaginase, and dexamethasone (VDCLP) regimen and found that doxorubicin displayed surprising inhibitory effects on *EP300-ZNF384* positive cells. Doxorubicin is an anthracycline antibiotic used in a wide range of cancers, including carcinomas, sarcomas, and hematological cancers [44]. Doxorubicin reportedly intercalates with their aglycone moieties between neighboring DNA base pairs, ultimately ceasing the replication and transcription of DNA [45]. Given its high affinity for DNA, doxorubicin may interrupt the growth of *EP300-ZNF384*-positive leukemia cells in vitro and in vivo (Fig. 6). Previous transcriptome profiling studies have reported that the DNA repair pathway is significantly decreased in *EP300-ZNF384* positive patients [8]. It is likely that doxorubicin exerts a therapeutic effect by inhibiting the transcription of *IL3RA* and weakening the DNA damage repair process. Our results indicated that B-ALL patients with *EP300-ZNF384* may benefit from chemotherapy regimens that include anthracyclines. Furthermore, an optimized chemotherapy protocol, including high-dose and intensive delivery of doxorubicin, may achieve better outcomes in patients with *EP300-ZNF384*. Collectively, our findings indicate that targeted therapies involving anthracyclines and CD123-targeted agents may produce a favorable outcome in patients with *EP300-ZNF384* and could be developed as a standard treatment for such cases.

Notably, this study has limitations that can be overcome in further research. First, we did not explain why *EP300-ZNF384* positive B-ALL cells are selectively sensitive to doxorubicin; thus, further research should investigate the mechanism of doxorubicin action on *EP300-ZNF384* positive B-ALL cells. Moreover, we did not evaluate the anti-leukemic role of targeting CD123 in *EP300-ZNF84* positive B-ALL; thus, further research should explore therapeutic potential of CD123-targeted therapies including CAR-engineered T cells, neutralizing antibodies, and IL3RA antibodies coupled to toxins. Further research should also attempt to explore the regulatory mechanism of *EP300-ZNF384* on *IL3* expression.

### Electronic supplementary material

Below is the link to the electronic supplementary material.

Supplementary Material 1

Supplementary Material 2

Supplementary Material 3: Fig. 1 EP300-ZNF384 promotes the expression of IL3RA in both B and myeloid leukemia cells. PLVX eukaryotic expression vectors encoding *EP*300-*ZNF384* and wild-type *ZNF384* were transfected into the acute B lymphoblastic leukemia (B-ALL) cell line BALL-1 (A), the Burkitt’s lymphoma cell line Daudi (B), and the acute myeloid leukemia cell line KG-1α (C) by lentivirus-mediated gene transfer. Total RNA was isolated from the transfectants, *IL3RA* expression was quantified by reverse-transcription quantitative PCR (RT-qPCR) in relative to *ACTB* expression. *P < 0.05, **P < 0.01, ***P < 0.001, ns, no significance. (D) Surface expression of CD123 was evaluated by flow cytometry on B-ALL cells with the empty vector, *EP300-ZNF384* fusion gene, and *ZNF384*. The proportion of CD123-positive cells and the median fluorescence intensity of CD123 was quantified. **P < 0.01, ***P < 0.001. Fig. 2 IL3RA plays an important role in EP300-ZNF384 induced cell proliferation and STAT5 activation with or without IL-3. (A) Immunoblot analysis of pSTAT5 and STAT5 in empty vector, *EP300-ZNF384*-, or *ZNF384*-expressing NALM-6 cells with or without IL-3. (B) sh*IL3RA* vectors (sh*IL3RA*-1, sh*IL3RA*-2, sh*IL3RA*-3, sh*IL3RA*-3 and empty vector) were transfected into NALM-6 cells. The knockdown efficiency was evaluated by reverse transcription quantitative PCR (RT-qPCR). **P < 0.01, ***P < 0.001, ns, no significance. (C) Sh*IL3RA* expressing NALM-6 cells were subjected to a colony-forming cell assay in the presence of IL-3 (10 ng/mL) or doxycycline (Dox, 2 µg/mL) or the combination of IL-3 with Dox. Quantitative analysis of colony size (diameter) and colony number was presented. *P < 0.05, ***P < 0.001, ns, no significance. (D) Sh*IL3RA*-4-expressing NALM-6 cells were transfected with *EP300-ZNF384* expressing vector. Cells were collected and RT-PCR was performed to assess the expression of *EP300-ZNF384* and *IL3RA*. (E) Cells derived from D were subjected to immune blot analysis to determine the expression of *EP300-ZNF384* and the phosphorylation of STAT5 in response to IL-3 treatment (10 ng/mL). Fig. 3 EP300-ZNF384 promotes the expression of CD123 on the cell membrane and the secretion of IL-3 in B precursor cells. Statistical analysis of surface CD123 expression on B precursor cells derived from 5 healthy volunteers with the empty vector, *EP300-ZNF384* fusion gene, and *ZNF384*. (A) The proportion of CD123-positive cells and (B) the median fluorescence intensity of CD123 were quantified. Data represent the mean ± SD. ns, no significance. (C) The expression pattern of *IL3* in hematopoiesis was presented. (D) CD19-positive cells were enriched with magnetic beads from PBMCs of healthy volunteers. Empty vector, *EP300-ZNF384-*, and *ZNF384*-expressing vectors were transfected into CD19-positive cells by lentivirus-mediated gene transfer. After 48 h, cells were collected for reverse transcription quantitative PCR assay to assess the transcription of *IL3*. Data represents mean ± SD. *P < 0.05, **P < 0.01, ***P < 0.001, ns, no significance. (E) Empty vector-, *EP300-ZNF384*-, and *ZNF384*-expressing vectors were respectively transfected into healthy volunteer derived CD19-positive cells in serum-free media by lentivirus-mediated gene transfer. Culture media was collected and IL-3 secretion was determined by ELISA. (*n* = 7) *P < 0.05, ns, no significance. Fig. 4 Doxorubicin specifically sensitized *EP300-ZNF384*-expressing B-ALL cells. The empty vector-, *EP300-ZNF384-* and *ZNF384*-expressing NALM-6 cells were treated with increasing doses of doxorubicin (A), dexamethasone (B), pegaspargase (C), and vincristine (D) for 48 h. Cell viability was evaluated using the CCK-8 assay. Fig. 5 Doxorubicin inhibited the infiltration of *EP300-ZNF384*-expressing B-ALL cells in bone marrow. Flow cytometry dot plots illustrating the infiltration of empty vector or *EP300-ZNF384* or *ZNF384*-expressing NALM-6 cells in the bone marrow of mice in response to doxorubicin treatment (*n* = 7).

## Data Availability

No datasets were generated or analysed during the current study.

## Abbreviations

B-ALL: Acute B lymphoblastic leukemia
MPAL: Mixed phenotype acute leukemia
IL-3: Interleukin 3
JAK/STAT: Janus kinase/signal transducers and activators of transcription
HSC: Hematopoietic stem cell
PBMCs: Peripheral blood mononuclear cells
CDX: Cell line-derived xenograft
CFC: Methylcellulose-based colony forming cell
FC: Fold change
MSigDB: Molecular Signatures Database
BM: Bone marrow
FBS: Fetal bovin serum
MNCs: Mononucleated cells
ShRNA: Short hairpin RNA
SDS-PAGE: Sodium dodecyl sulfate-polyacrylamide gel
Dox: Doxycycline
DEGs: Differentially expressed genes
GSEA: Gene set enrichment analysis
ZNF384: Wild-type ZNF384
Vector: Empty vector
RT-qPCR: Reverse transcription- quantitative polymerase chain reaction
ChIP: Chromatin immunoprecipitation
PI: Propidium iodide
HSCT: Hematopoietic stem cell transplantation
RPKM: Million mapped reads
GTF file: Gene annotation format file
HE: Hematoxylin and eosin

## References

[1] Gocho Y, Kiyokawa N, Ichikawa H, Nakabayashi K, Osumi T, Ishibashi T (2015). A novel recurrent EP300-ZNF384 gene fusion in B-cell precursor acute lymphoblastic leukemia. Leukemia.

[2] Alexander TB, Gu Z, Iacobucci I, Dickerson K, Choi JK, Xu B (2018). The genetic basis and cell of origin of mixed phenotype acute leukaemia. Nature.

[3] Zhao X, Wang P, Diedrich JD, Smart B, Reyes N, Yoshimura S (2022). Epigenetic activation of the FLT3 gene by ZNF384 fusion confers a therapeutic susceptibility in acute lymphoblastic leukemia. Nat Commun.

[4] Chen X, Wang F, Zhang Y, Ma X, Cao P, Yuan L (2021). Fusion gene map of acute leukemia revealed by transcriptome sequencing of a consecutive cohort of 1000 cases in a single center. Blood Cancer J.

[5] Hirabayashi S, Ohki K, Nakabayashi K, Ichikawa H, Momozawa Y, Okamura K (2017). ZNF384-related fusion genes define a subgroup of childhood B-cell precursor acute lymphoblastic leukemia with a characteristic immunotype. Haematologica.

[6] Yamamoto H, Hayakawa F, Yasuda T, Odaira K, Minamikawa Y, Tange N (2019). ZNF384-fusion proteins have high affinity for the transcriptional coactivator EP300 and aberrant transcriptional activities. FEBS Lett.

[7] Li JF, Dai YT, Lilljebjorn H, Shen SH, Cui BW, Bai L (2018). Transcriptional landscape of B cell precursor acute lymphoblastic leukemia based on an international study of 1,223 cases. Proc Natl Acad Sci U S A.

[8] McClure BJ, Heatley SL, Kok CH, Sadras T, An J, Hughes TP (2018). Pre-B acute lymphoblastic leukaemia recurrent fusion, EP300-ZNF384, is associated with a distinct gene expression. Br J Cancer.

[9] Liu YF, Wang BY, Zhang WN, Huang JY, Li BS, Zhang M (2016). Genomic profiling of Adult and Pediatric B-cell Acute Lymphoblastic Leukemia. EBioMedicine.

[10] Yasuda T, Tsuzuki S, Kawazu M, Hayakawa F, Kojima S, Ueno T (2016). Recurrent DUX4 fusions in B cell acute lymphoblastic leukemia of adolescents and young adults. Nat Genet.

[11] Qian M, Zhang H, Kham SK, Liu S, Jiang C, Zhao X (2017). Whole-transcriptome sequencing identifies a distinct subtype of acute lymphoblastic leukemia with predominant genomic abnormalities of EP300 and CREBBP. Genome Res.

[12] Yaguchi A, Ishibashi T, Terada K, Ueno-Yokohata H, Saito Y, Fujimura J (2017). EP300-ZNF384 fusion gene product up-regulates GATA3 gene expression and induces hematopoietic stem cell gene expression signature in B-cell precursor acute lymphoblastic leukemia cells. Int J Hematol.

[13] Huang S, Chen Z, Yu JF, Young D, Bashey A, Ho AD (1999). Correlation between IL-3 receptor expression and growth potential of human CD34 + hematopoietic cells from different tissues. Stem Cells.

[14] Lundberg K, Rydnert F, Greiff L, Lindstedt M (2014). Human blood dendritic cell subsets exhibit discriminative pattern recognition receptor profiles. Immunology.

[15] Taussig DC, Pearce DJ, Simpson C, Rohatiner AZ, Lister TA, Kelly G (2005). Hematopoietic stem cells express multiple myeloid markers: implications for the origin and targeted therapy of acute myeloid leukemia. Blood.

[16] Hercus TR, Dhagat U, Kan WL, Broughton SE, Nero TL, Perugini M (2013). Signalling by the betac family of cytokines. Cytokine Growth Factor Rev.

[17] Klein BK, Shieh JJ, Grabbe E, Li X, Welply JK, McKearn JP (2001). Receptor binding kinetics of human IL-3 variants with altered proliferative activity. Biochem Biophys Res Commun.

[18] Thomas JW, Baum CM, Hood WF, Klein B, Monahan JB, Paik K (1995). Potent interleukin 3 receptor agonist with selectively enhanced hematopoietic activity relative to recombinant human interleukin 3. Proc Natl Acad Sci U S A.

[19] Broughton SE, Dhagat U, Hercus TR, Nero TL, Grimbaldeston MA, Bonder CS (2012). The GM-CSF/IL-3/IL-5 cytokine receptor family: from ligand recognition to initiation of signaling. Immunol Rev.

[20] Liu K, Zhu M, Huang Y, Wei S, Xie J, Xiao Y (2015). CD123 and its potential clinical application in leukemias. Life Sci.

[21] Testa U, Pelosi E, Frankel A (2014). CD 123 is a membrane biomarker and a therapeutic target in hematologic malignancies. Biomark Res.

[22] Shi M, Su RJ, Parmar KP, Chaudhry R, Sun K, Rao J (2019). CD123: a novel biomarker for diagnosis and treatment of Leukemia. Cardiovasc Hematol Disord Drug Targets.

[23] Al-Mawali A, Gillis D, Lewis I (2016). Immunoprofiling of leukemic stem cells CD34+/CD38-/CD123 + delineate FLT3/ITD-positive clones. J Hematol Oncol.

[24] Testa U, Riccioni R, Militi S, Coccia E, Stellacci E, Samoggia P (2002). Elevated expression of IL-3Ralpha in acute myelogenous leukemia is associated with enhanced blast proliferation, increased cellularity, and poor prognosis. Blood.

[25] Wittwer NL, Brumatti G, Marchant C, Sandow JJ, Pudney MK, Dottore M (2017). High CD123 levels enhance proliferation in response to IL-3, but reduce chemotaxis by downregulating CXCR4 expression. Blood Adv.

[26] Das N, Gupta R, Gupta SK, Bakhshi S, Malhotra A, Rai S (2020). A real-world perspective of CD123 expression in Acute Leukemia as Promising Biomarker to Predict Treatment Outcome in B-ALL and AML. Clin Lymphoma Myeloma Leuk.

[27] Wang YZ, Qin YZ, Chang Y, Yuan XY, Chen WM, He LL (2022). Immunophenotypic characteristics of ZNF384 rearrangement compared with BCR-ABL1, KMT2A rearrangement, and other adult B-cell precursor acute lymphoblastic leukemia. Cytometry B Clin Cytom.

[28] Dobin A, Davis CA, Schlesinger F, Drenkow J, Zaleski C, Jha S (2013). STAR: ultrafast universal RNA-seq aligner. Bioinformatics.

[29] Trapnell C, Williams BA, Pertea G, Mortazavi A, Kwan G, van Baren MJ (2010). Transcript assembly and quantification by RNA-Seq reveals unannotated transcripts and isoform switching during cell differentiation. Nat Biotechnol.

[30] Gu Z, Churchman M, Roberts K, Li Y, Liu Y, Harvey RC (2016). Genomic analyses identify recurrent MEF2D fusions in acute lymphoblastic leukaemia. Nat Commun.

[31] Jing Y, Li YF, Wan H, Liu DH (2020). Detection of EP300-ZNF384 fusion in patients with acute lymphoblastic leukemia using RNA fusion gene panel sequencing. Ann Hematol.

[32] Lamble AJ, Eidenschink Brodersen L, Alonzo TA, Wang J, Pardo L, Sung L (2022). CD123 expression is Associated with High-Risk Disease characteristics in Childhood Acute myeloid leukemia: a Report from the children’s Oncology Group. J Clin Oncol.

[33] Stoeckle C, Simon HU (2013). CD8(+) T cells producing IL-3 and IL-5 in non-IgE-mediated eosinophilic diseases. Allergy.

[34] Miyauchi J, Kelleher CA, Yang YC, Wong GG, Clark SC, Minden MD (1987). The effects of three recombinant growth factors, IL-3, GM-CSF, and G-CSF, on the blast cells of acute myeloblastic leukemia maintained in short-term suspension culture. Blood.

[35] Eder M, Ottmann OG, Hansen-Hagge TE, Bartram CR, Falk S, Gillis S (1992). In vitro culture of common acute lymphoblastic leukemia blasts: effects of interleukin-3, interleukin-7, and accessory cells. Blood.

[36] Agger K, Miyagi S, Pedersen MT, Kooistra SM, Johansen JV, Helin K (2016). Jmjd2/Kdm4 demethylases are required for expression of Il3ra and survival of acute myeloid leukemia cells. Genes Dev.

[37] Okamoto K, Imamura T, Tanaka S, Urata T, Yoshida H, Shiba N (2023). The Nup98::Nsd1 fusion gene induces CD123 expression in 32D cells. Int J Hematol.

[38] Gill S, Tasian SK, Ruella M, Shestova O, Li Y, Porter DL (2014). Preclinical targeting of human acute myeloid leukemia and myeloablation using chimeric antigen receptor-modified T cells. Blood.

[39] Busfield SJ, Biondo M, Wong M, Ramshaw HS, Lee EM, Ghosh S (2014). Targeting of acute myeloid leukemia in vitro and in vivo with an anti-CD123 mAb engineered for optimal ADCC. Leukemia.

[40] Mani R, Goswami S, Gopalakrishnan B, Ramaswamy R, Wasmuth R, Tran M (2018). The interleukin-3 receptor CD123 targeted SL-401 mediates potent cytotoxic activity against CD34(+)CD123(+) cells from acute myeloid leukemia/myelodysplastic syndrome patients and healthy donors. Haematologica.

[41] Han L, Jorgensen JL, Brooks C, Shi C, Zhang Q, Nogueras Gonzalez GM (2017). Antileukemia Efficacy and mechanisms of Action of SL-101, a Novel Anti-CD123 antibody conjugate, in Acute myeloid leukemia. Clin Cancer Res.

[42] Angelova E, Audette C, Kovtun Y, Daver N, Wang SA, Pierce S (2019). CD123 expression patterns and selective targeting with a CD123-targeted antibody-drug conjugate (IMGN632) in acute lymphoblastic leukemia. Haematologica.

[43] Malard F, Mohty M (2020). Acute lymphoblastic leukaemia. Lancet.

[44] Minotti G, Menna P, Salvatorelli E, Cairo G, Gianni L (2004). Anthracyclines: molecular advances and pharmacologic developments in antitumor activity and cardiotoxicity. Pharmacol Rev.

[45] Sarkar R, Patra U, Lo M, Mukherjee A, Biswas A, Chawla-Sarkar M (2020). Rotavirus activates a noncanonical ATM-Chk2 branch of DNA damage response during infection to positively regulate viroplasm dynamics. Cell Microbiol.

