# Supplementary figures and images for: EP300-ZNF384 transactivates IL3RA to promote the progression of B-cell acute lymphoblastic leukemia
Source: Cell Commun Signal. 2024 Apr 2;22:211. doi: 10.1186/s12964-024-01596-9 (PMC10986138; doi:10.1186/s12964-024-01596-9)

Full uncropped Gels and Blots images

Fig. 1F

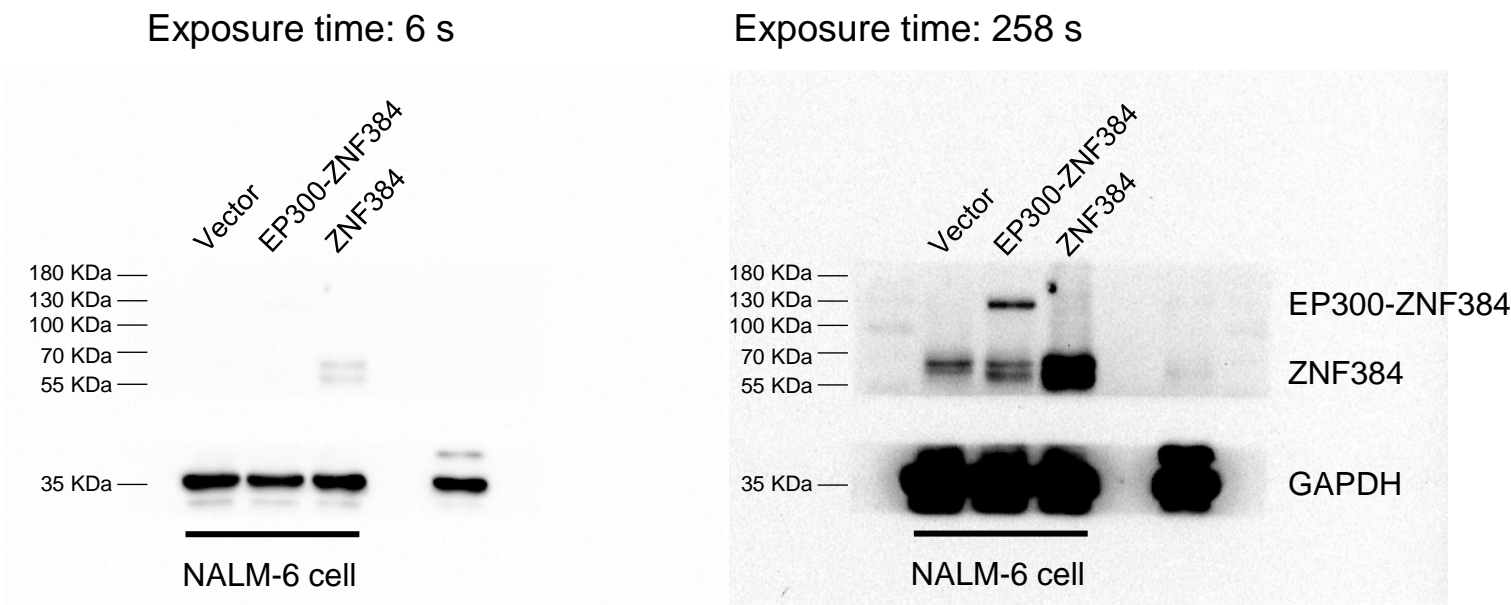

Fig. 4G

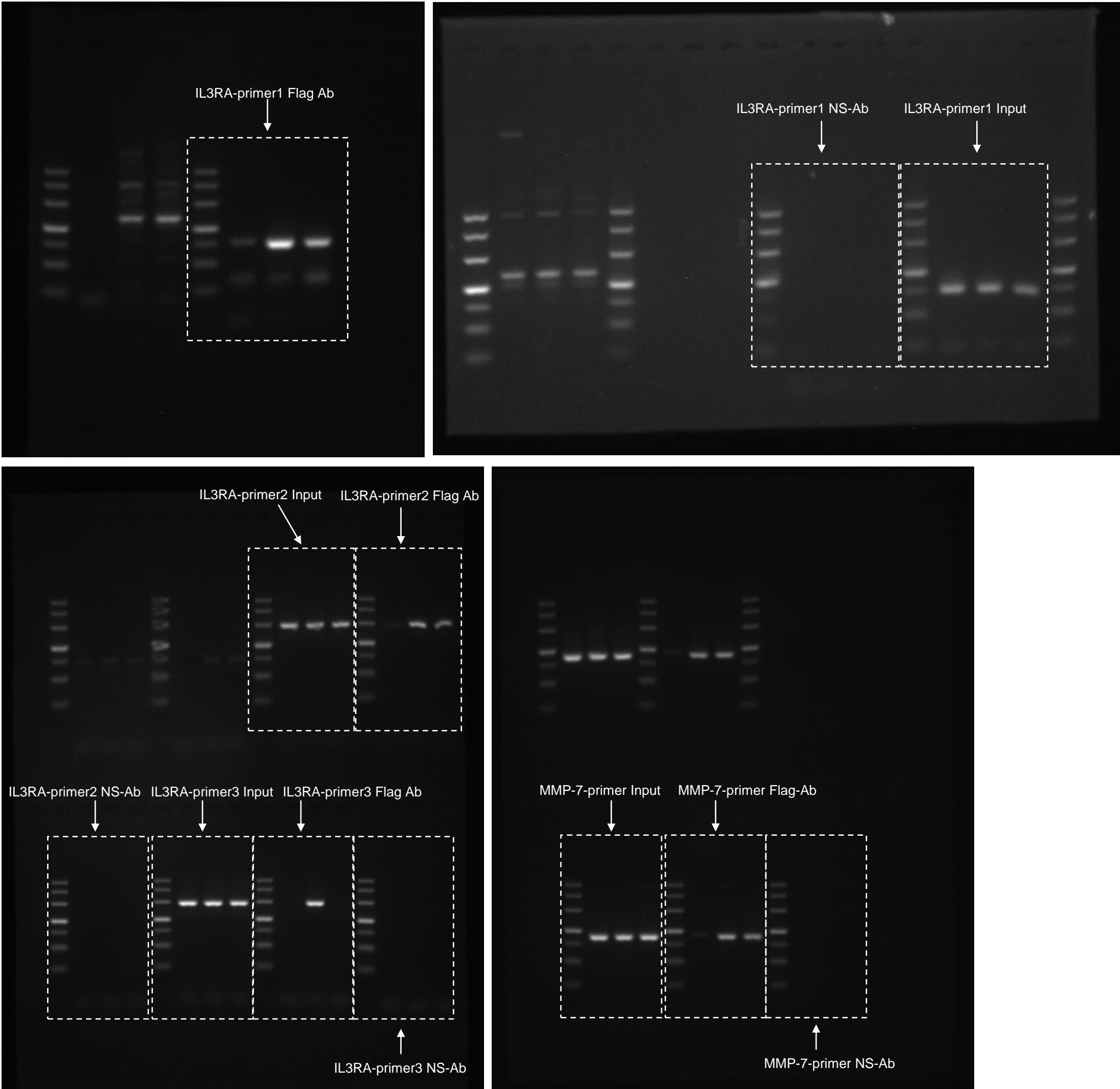

Fig. S2A

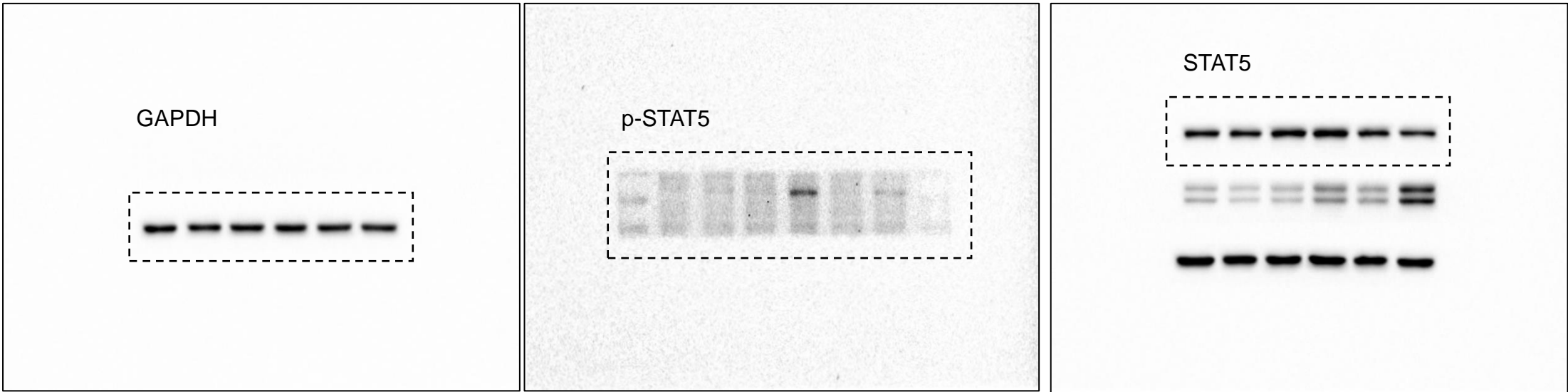

Fig. S2D

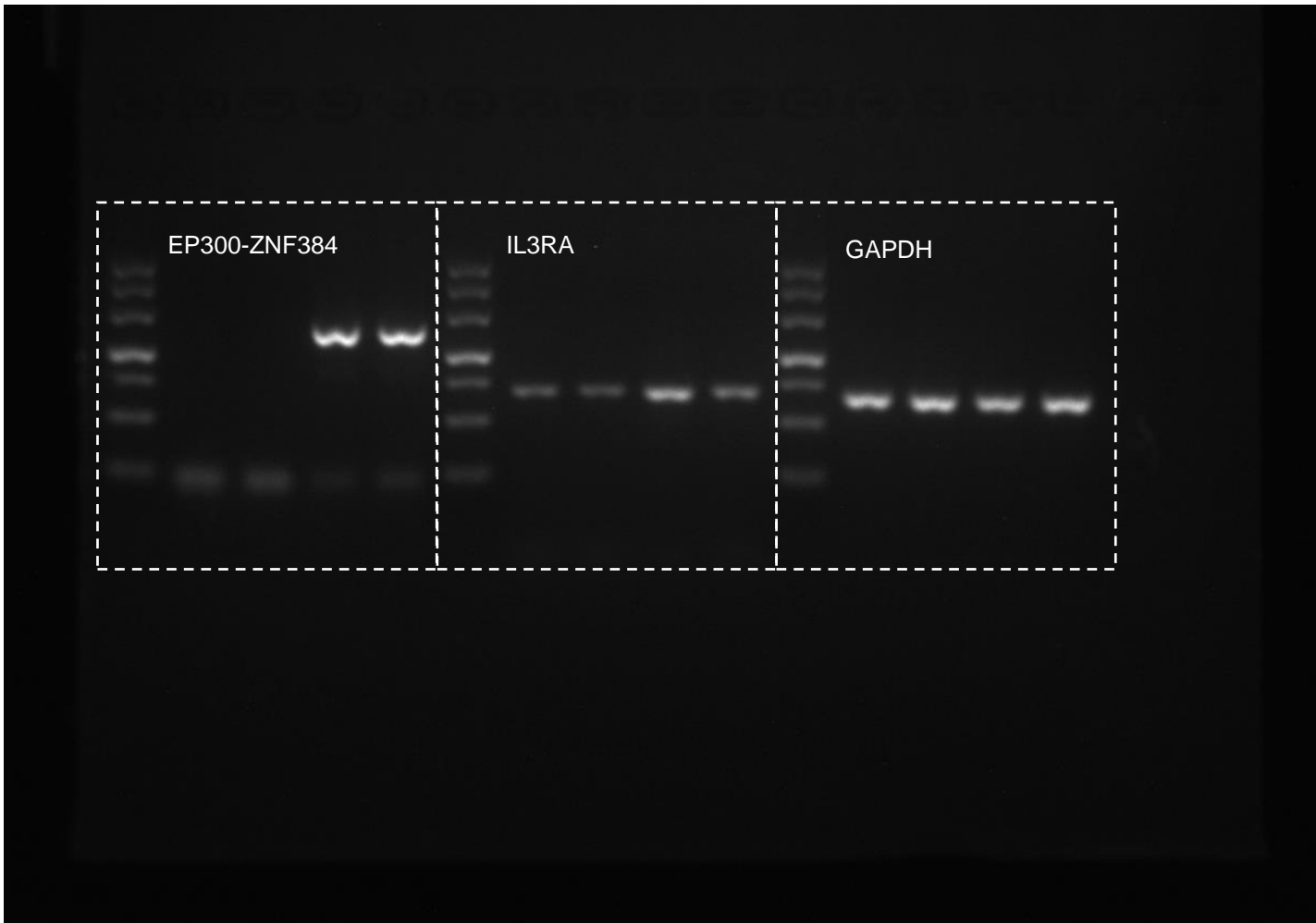

Fig. S2E

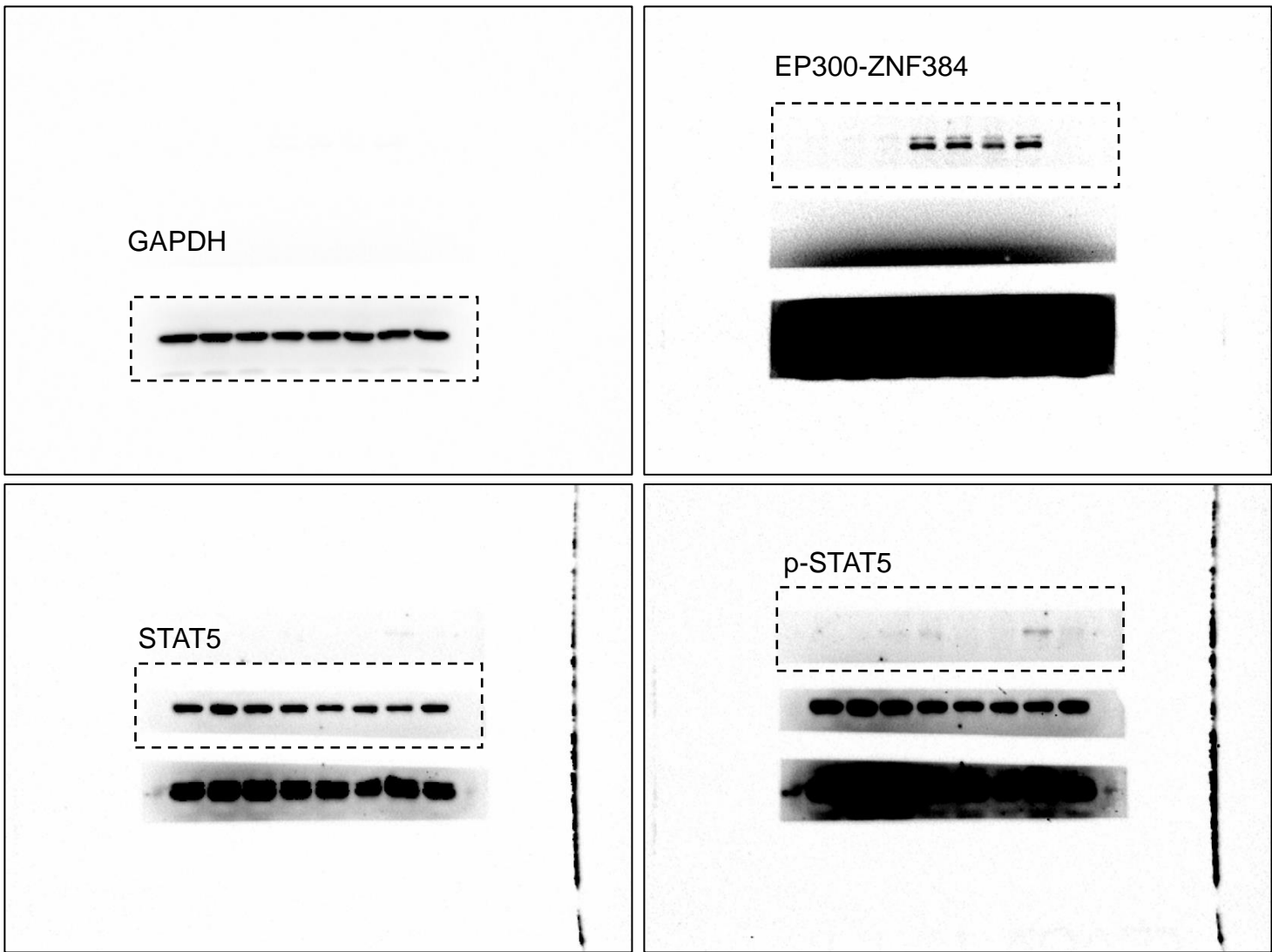

Supplement: Supplementary file 2 — Supplementary Material 2 [file 12964_2024_1596_MOESM2_ESM.pdf]
