# Supplementary material for: EP300-ZNF384 transactivates IL3RA to promote the progression of B-cell acute lymphoblastic leukemia
Source: Cell Commun Signal. 2024 Apr 2;22:211. doi: 10.1186/s12964-024-01596-9 (PMC10986138; doi:10.1186/s12964-024-01596-9)

Fig.S1 *EP300-ZNF384* promotes the expression of *IL3RA* in both B and myeloid leukemia cells.

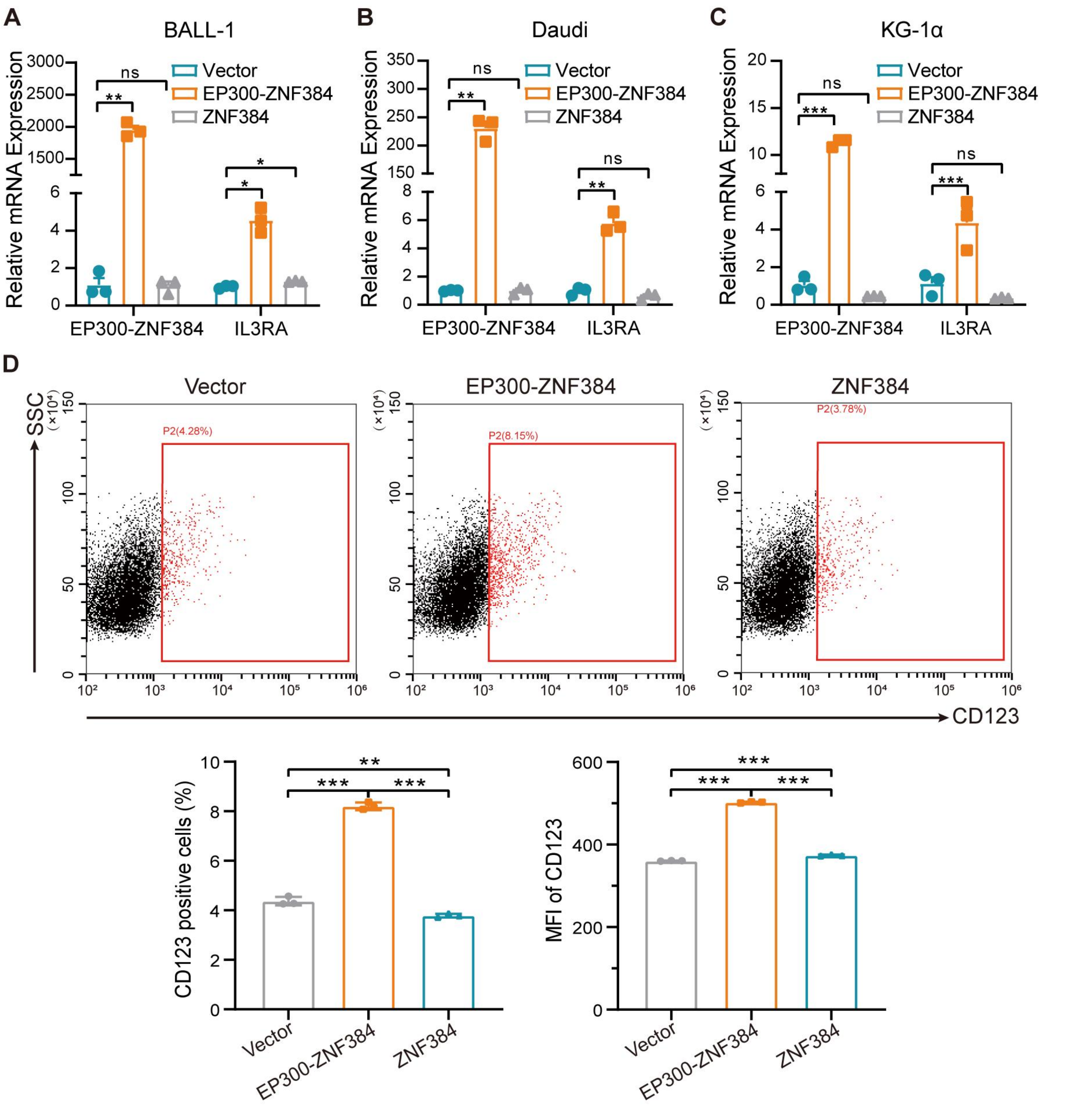

Fig. S2 *IL3RA* plays an important role in *EP300-ZNF384* induced cell proliferation and STAT5 acitivation with or without IL-3.

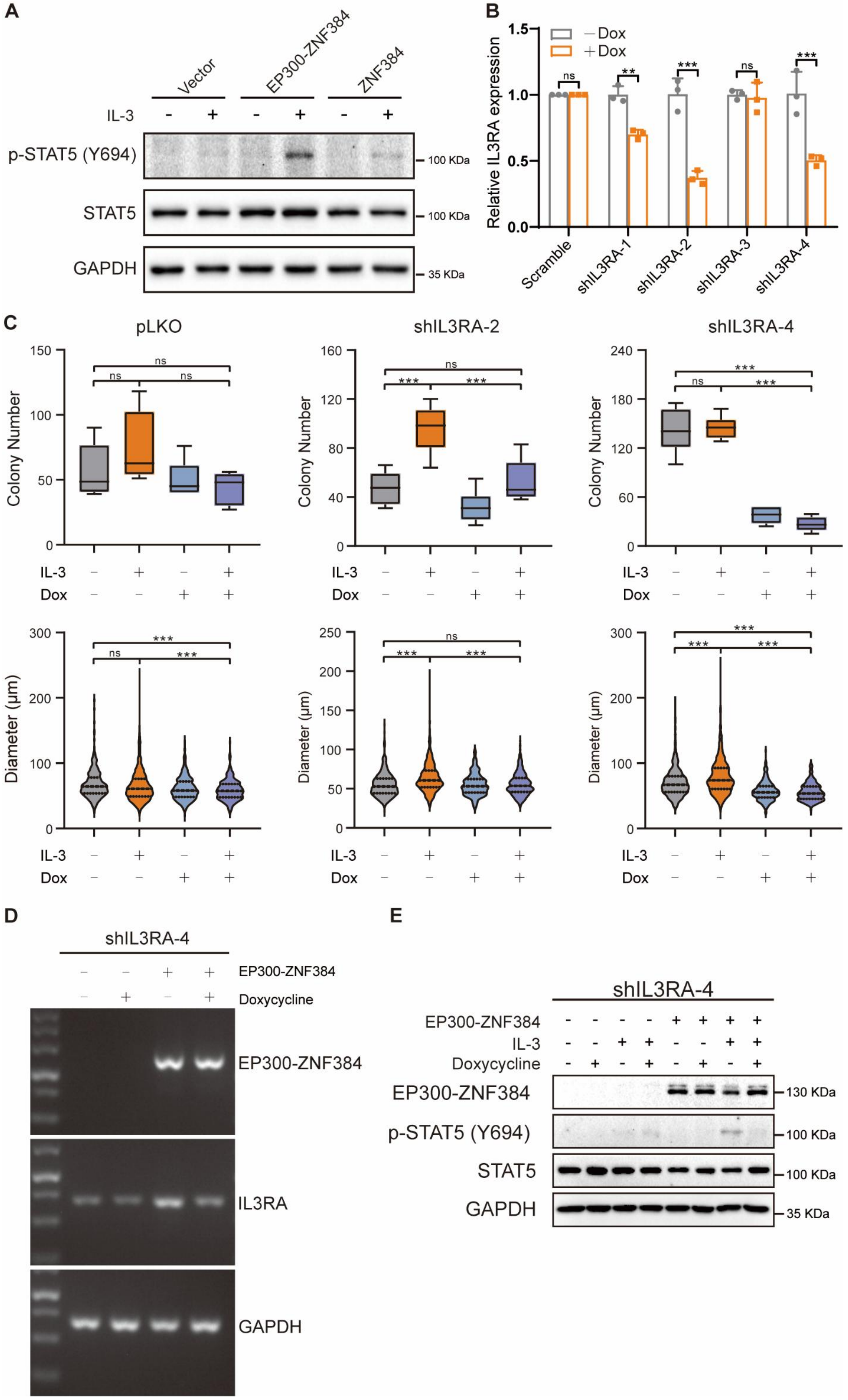

Fig. S3 *EP300-ZNF384* promotes the expression of CD123 on the cell membrane and the secretion of IL-3 in B precursor cells.

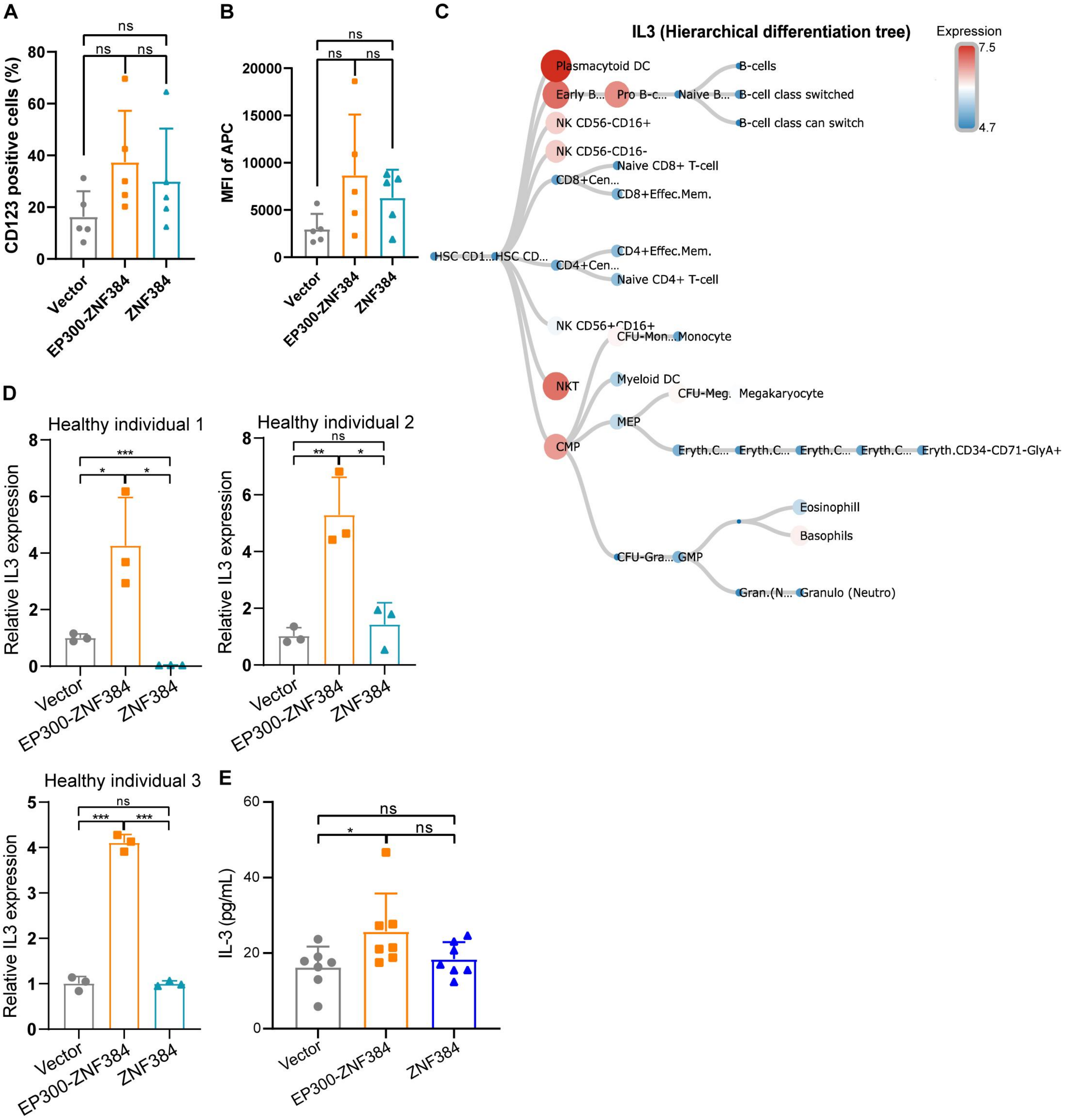

Fig. S4 Doxorubicin specifically sensitized *EP300-ZNF384*-expressing B-ALL cells.

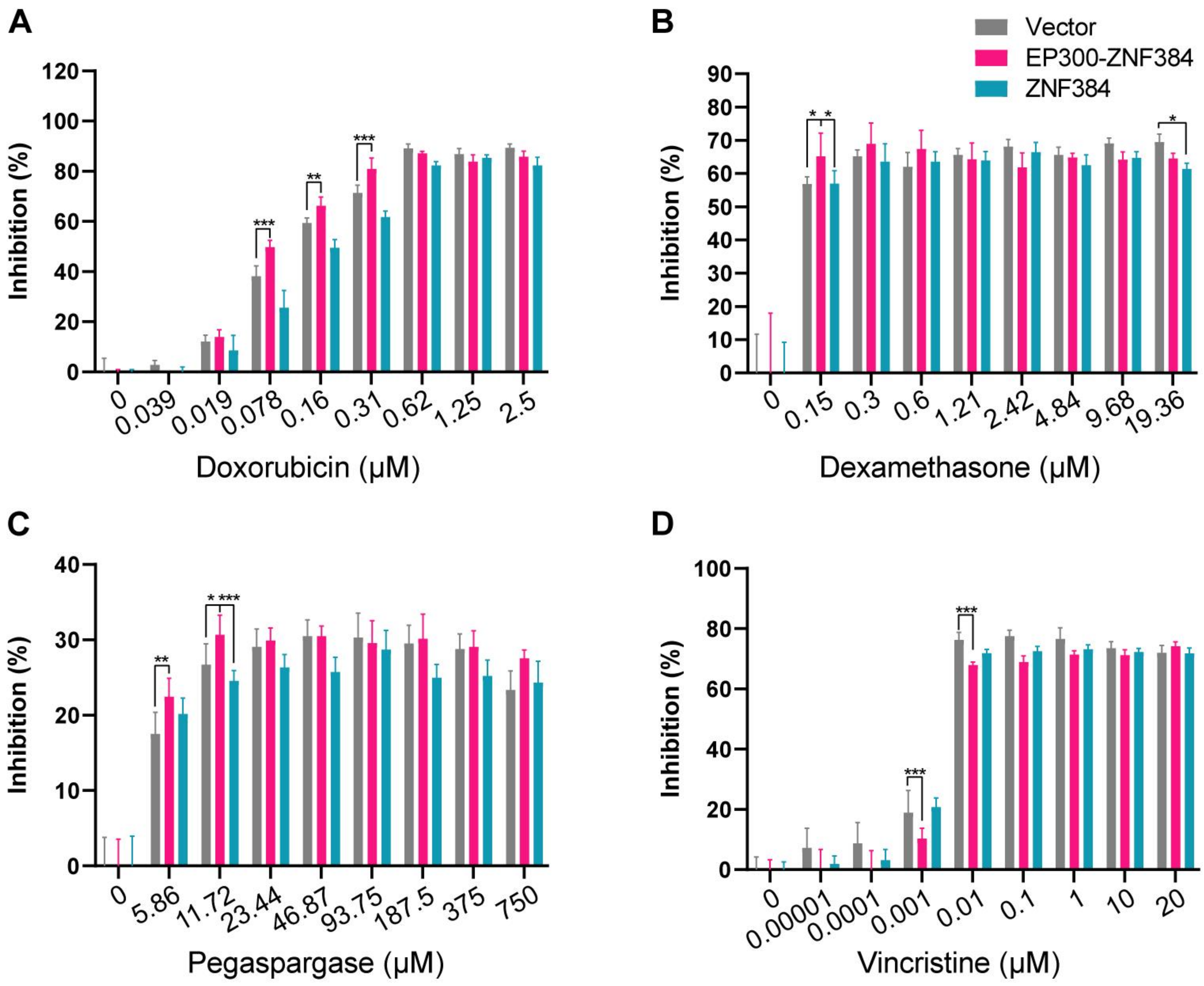

Fig. S5 Doxorobicin inhibited the infiltration of *EP300-ZNF384*-expressing B-ALL cells in bone marrow.

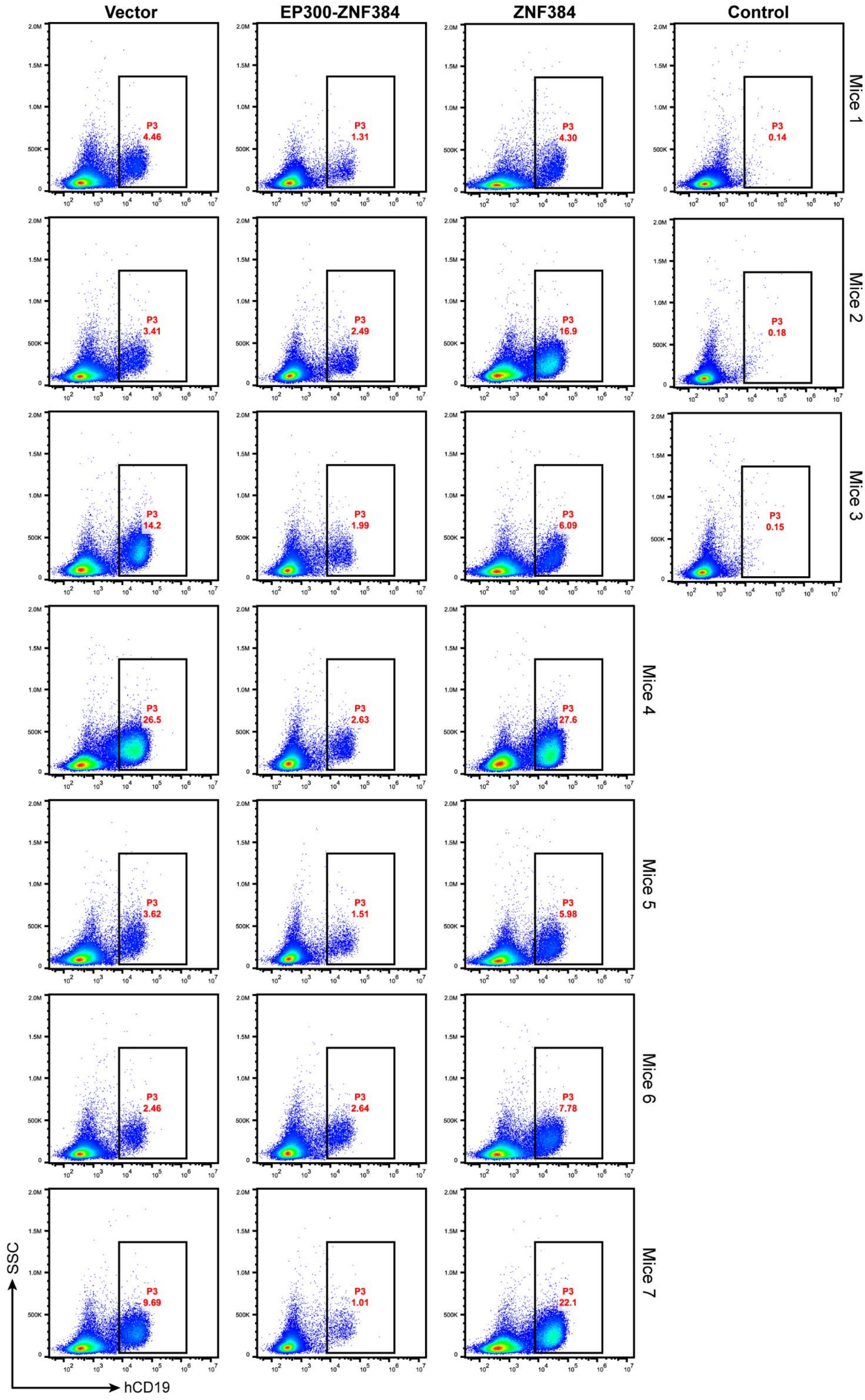

Supplement: Supplementary file 3 — Supplementary Material 3: Fig. 1 EP300-ZNF384 promotes the expression of IL3RA in both B and myeloid leukemia cells. PLVX eukaryotic expression vectors encoding EP300-ZNF384 and wild-type ZNF384 were transfected into the acute B lymphoblastic leukemia (B-ALL) cell line BALL-1 (A), the Burkitt’s lymphoma cell line Daudi (B), and the acute myeloid leukemia cell line KG-1α (C) by lentivirus-mediated gene transfer. Total RNA was isolated from the transfectants, IL3RA expression was quantified by reverse-transcription quantitative PCR (RT-qPCR) in relative to ACTB expression. *P < 0.05, **P < 0.01, ***P < 0.001, ns, no significance. (D) Surface expression of CD123 was evaluated by flow cytometry on B-ALL cells with the empty vector, EP300-ZNF384 fusion gene, and ZNF384. The proportion of CD123-positive cells and the median fluorescence intensity of CD123 was quantified. **P < 0.01, ***P < 0.001. Fig. 2 IL3RA plays an important role in EP300-ZNF384 induced cell proliferation and STAT5 activation with or without IL-3. (A) Immunoblot analysis of pSTAT5 and STAT5 in empty vector, EP300-ZNF384-, or ZNF384-expressing NALM-6 cells with or without IL-3. (B) shIL3RA vectors (shIL3RA-1, shIL3RA-2, shIL3RA-3, shIL3RA-3 and empty vector) were transfected into NALM-6 cells. The knockdown efficiency was evaluated by reverse transcription quantitative PCR (RT-qPCR). **P < 0.01, ***P < 0.001, ns, no significance. (C) ShIL3RA expressing NALM-6 cells were subjected to a colony-forming cell assay in the presence of IL-3 (10 ng/mL) or doxycycline (Dox, 2 µg/mL) or the combination of IL-3 with Dox. Quantitative analysis of colony size (diameter) and colony number was presented. *P < 0.05, ***P < 0.001, ns, no significance. (D) ShIL3RA-4-expressing NALM-6 cells were transfected with EP300-ZNF384 expressing vector. Cells were collected and RT-PCR was performed to assess the expression of EP300-ZNF384 and IL3RA. (E) Cells derived from D were subjected to immune blot analysis to determine the ex [file 12964_2024_1596_MOESM3_ESM.pdf]
